# Silicon amendment induces synergistic plant defense mechanism against pink stem borer (*Sesamia inferens* Walker.) in finger millet (*Eleusine coracana* Gaertn.)

**DOI:** 10.1038/s41598-020-61182-0

**Published:** 2020-03-06

**Authors:** Kundansing Rajpalsing Jadhao, Anuradha Bansal, Gyana R. Rout

**Affiliations:** 1 Department of Agricultural Biotechnology, College of Agriculture, Odisha University of Agriculture & Technology, Bhubaneswar, 751003 Odisha India; 2 Biointeractions and Crop Protection, Rothamsted Research, West Common, Harpenden, Hertfordshire, AL5 2JQ United Kingdom

**Keywords:** Agricultural genetics, Plant molecular biology

## Abstract

Silicon (Si) uptake and accumulation in plants can mitigate various biotic stresses through enhanced plant resistance against wide range of herbivores. But the role of silicon in defense molecular mechanism still remains to be elucidated in finger millet. In the present study, we identified three silicon transporter genes viz. *EcLsi1*, *EcLsi2*, and *EcLsi6* involved in silicon uptake mechanism. In addition, the study also identified and characterized ten different Si transporters genes from finger millet through transcriptome assembly. The phylogenetic study revealed that *EcLsi1* and *EcLsi6 *are homologs while *EcLsi2* and *EcLsi3 *form another pair of homologs. *EcLsi1* and *EcLsi6* belong to family of NIP2s (Nod26-like major intrinsic protein), *bona fide* silicon transporters, whereas *EcLsi2* and *EcLsi3*, an efflux Si transporter, belong to an uncharacterized anion transporter family having a significant identity with putative arsB transporter proteins. Further, the phylogenetic and topology analysis suggest that *EcLsi1* and *EcLsi2* co-evolved during evolution while, *EcLsi2* and *EcLsi3* are evolved from either *EcLsi1* and/or *EcLsi6* by fusion or duplication event. Moreover, these silicon transporters are predicted to be localized in plasma membrane, but their structural differences indicate that they might have differences in their silicon uptake ability. Silicon amendment induces the synergistic defense mechanism by significantly increasing the transcript level of silicon transporter genes (*EcLsi1*, *EcLsi2* and *EcLsi6*) as well as defense hormone regulating genes (*EcSAM*, *EcPAL* and *EcLOX*) at 72 hpi (hours of post infestation) in both stem and roots compared to non-silicon treated plants against pink stem borer in finger millet plants. This study will help to understand the molecular defense mechanism for developing strategies for insect pest management.

## Introduction

Finger millet (*Eleusine coracana* Gaertn.) is the most important cereal crop among small millets after sorghum and pearl millet and is grown for food and fodder in India. It is the third most widely cultivated millet crop in the semi-arid tropical and sub-tropical regions worldwide^1,2^. Finger millet is a sustainable food of poor people in rural areas and is also appreciated by urban populations for its nutritional quality as it provides fair amount of proteins, minerals, calcium and vitamins compared to other cereals. Still, finger millet is neglected in developing countries where food security is the major for the growing population^3^. Along with its nutritional quality to solve dietary issues in rural areas, finger millet is also considered as one of the hardiest crop to mitigate the effects of drought and numerous biotic stresses^4^. Although, it is being affected by numerous insects and pests, pink stem borer *Sesamia inferens* Walker (Noctuidae; Lepidoptera), is one of the major biotic constraints for crop production in finger millet growing countries^5^. Pink stem borer is a polyphagous pest which feeds on wide range of grasses including finger millet. It is widely recorded in India and different parts of South Asia^5^. Despite the economic importance of this borer, there is lack of a good pest management strategy. Currently, conventional cultural practices are preferred for suppressing the pest population during larval and pupal stages with some use of pesticides in more severe condition^5^. However, *S.inferens* has become less sensitive to many recommended pesticides in recent years^6^. Therefore, integrated pest management strategies recommend the combination of preventive measures using stem borer resistant/tolerant cultivars for planting, avoiding the stem borer favored conditions for planting and encouragement of indigenous parasitoids and natural enemies^7^. But there is an urgent need to develop effective and ecologically sound alternative method to control stem borer infestation in finger millet to maintain the high productivity. Silicon (Si)is now being recognized as a sustainable alternative to provide resistance against difference biotic and abiotic stresses which ultimately influence the crop production worldwide^8,9^. Although Si is not recognized as an essential element required for plant nutrition^10^, its beneficial effects against different insect pests has been demonstrated in many plant species^8^. For instance, it has been demonstrated that Si increases tolerance against all types of borers including stem borer in various cereal crops^11^. Hence, it was reported that Si is a “silver bullet” and a potential alternative for biotic stress management in different crop species^12^. It is widely known that Si creates a physical barrier by silicification of plant parts as part of defense mechanisms against insect pests. Silicon acts as an elicitor of systemic stress signals to produce the effective defense compounds against herbivores by regulating the defense related phytohormone pathways^13^. Still plant defense is a complex process and it can vary with feeding strategies of insect pests^14^. The phytohormones like salicylic acid (SA), jasmonic acid (JA) and ethylene (ET) are the first line of defense in enhancing the plant responses to various herbivores^15^. JA and SA regulate defenses against herbivores, particularly JA controls cell-content-feeding and tissue-chewing insects^16,17^ while SA and JA together regulate the defense against phloem-feeding insects^18^. It has been shown recently that Si induces JA-mediated defense responses against insects and pests^19^, indicating that Si can enhance the plant resistance against herbivores by regulating the defense responsive phytohormone pathways. Si also induces the priming process in host plant by sensitizing and preparing the plant for quick responses against the future herbivore attacks^20–22^. The mechanism of uptake of Si varies amongst different plant species and apparently depends on the presence of specific Si transporters. The first report on identification of Si transporters in plants was in rice^13^. Genes involved in Si uptake and distribution in numbers of plant species like barley, maize, pumpkin, wheat, cucumber and horsetail millet have now been identified^23,24^. These transporters, namely *Lsi1* and *Lsi6*, are Si-influx transporters belonging to the aquaporin family and are mainly involved in Si distribution in root and shoot tissues^25^. *Lsi2* belongs to the putative anion transporter and primarily expressed in the endodermis of roots, is proton driven and it works as a Si/H + antiporter^26^. However, these transporters do not show polar localization in barley and maize^27^. Hence, the silicon uptake ability of *Lsi1* and *Lsi2* varies greatly in different plant species in comparison to rice^28^. Therefore, the aim of the present study is to isolate, identify, and characterize the silicon transporter genes in finger millet and study their expression to dissect the molecular defense mechanism and interaction with phytohormones against pink stem borer in finger millet plant.

## Results

### Isolation and identification of Si transporter genes

**Figure 1:**
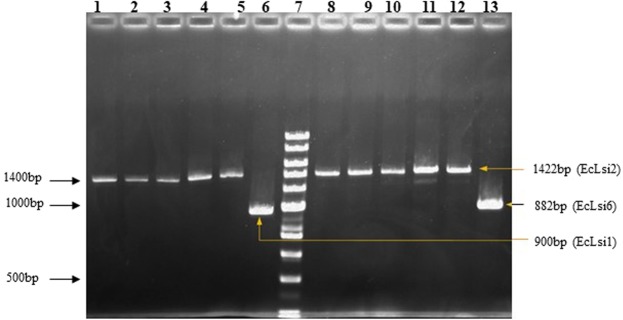
Amplification of silicon transporter genes viz. *EcLsi1*, *EcLsi2* and *Eclsi6 *from finger millet employing gene specific primers. 1–4: *EcLsi2* gene amplification from root tissue; 6: *EcLsi1* gene amplification; 7: Low range DNA ruler plus; 8–12: *EcLsi2* gene amplification from stem tissue; 13: *EcLsi6* gene amplification. Numbers on the right side of margin represents molecular weight marker DNA in base pairs (bps) and number on left side represents size of amplified product size in base pairs.

Isolation of *EcLsi1*, *EcLsi2* and *EcLsi6* CDS region from finger millet cv. Suvra employed PCR amplification using the gene specific oligonucleotides (Table S1**)**. The PCR amplification produced sharp and bright bands of 900 bp, 1422 bp and 882bp for *EcLsi1*, *EcLsi2* and *EcLsi6* respectively (Fig. 1). The CDS regions of all three isolated genes were subjected to BLASTn hit search for identification of the genes. The *EcLsi1 gene* showed maximum 91.04% identity with *Setaria italica* aquaporin NIP2-1 mRNA (Accession no. XM_004953810.4), followed by 90.92% identity with Sorghum *bicolor NOD*26-like major intrinsic protein (NIP2-1) mRNA, complete CDS (Accession no. EF373651.1.). The *EcLsi2* and *EcLsi6* showed maximum 88.60% and 92.72% identity with *Setaria italica* silicon efflux transporter *LSI2*, mRNA (accession no. XM_004985998.2) and *Setaria italica* aquaporin NIP2-2, mRNA (accession number. XM_004964985.4) respectively. The blast result indicates that *E.coracana* silicon transporter genes are orthologous to millet family member. After identification, the CDS sequences of *EcLsi1*, *EcLsi2* and *EcLsi6 *genes isolated from finger millet variety ‘Suvra’ were submitted to NCBI with the accession nos. MN517554, MN517555 and MN517556 (NCBI) respectively.

### Transcriptome assembly

To identify the homologous silicon transporter genes, the high-throughput ILLUMINA (Next Seq 500) paired-end reads transcriptome library of finger millet cv. ML-365 was retrieved from NCBI database bearing the accession number SRR4021829. The obtained library was further assembled to mine the transcriptionally active silicon transporter genes. The raw reads of library were cleaned for adapter contamination and filtered for low-quality reads with Q20 base parameter with the Trimmomatic program. Reads less than 20 bp length were removed from libraries before assembling. De-novo assembly of these high-quality reads was performed with Trinity assembler which produced 2,64,541 unigenes after assembly. CAP3 program was then employed to reduce the overlapping length with cut off parameter > 50 and specific overlap percent identity cutoff of > 95% similarity. After CAP3 assembly, a total of 2,39,594 unigenes were generated. The unigenes were further filtered for removing sequence duplicates. The redundant unigene sequences were removed using CD-HIT-EST (v4.6.1) tool with 95% identity threshold. The CD-HIT-EST assembly yielded 1,89,632 unigenes, generating mean sequence length of 888 nt and median sequence length of 535 nt (N50 = 1736) with GC-content of 45.81% (Table S2). The largest and shortest transcript/unigene lengths were 24966 nt and 201 nt respectively after assembly. These unigenes were further used for ORF prediction for CDS identification, functional annotation and other downstream analyses. Predicted CDSs from transcriptome were subjected to BLASTx search against non-redundant (nr) protein database of NCBI with an E-value cut-off of 10e^−6^. Total of 1440 plant silicon transporter genes available on NCBI were queried against the predicted CDSs, total 1117 (77.57%) number of complete core genes were detected and 1272 (88.33%) complete and partial genes were detected (Table S2). After the final annotation, 10 CDSs having the significant hit with silicon transporter genes were identified as variant silicon transporter genes of finger millet. The maximum similarity of 94.26% was observed with *Setaria italica* silicon efflux transporter LSI2 isoform X1 (accession number XP_004985708.1) and minimum 81.22% with *Panicum hallii* silicon efflux transporter *LSI2*-like (accession number XP_025817714.1) (Table S3). Out of 10 predicted variant silicon transporter genes, 5 were Silicon-efflux transporter (*EcLsi2*) genes, 4 were silicon-efflux transporter (*EcLsi3*) genes and 1 was NOD26-like major intrinsic protein (*EcLsi1*) gene (Table S3).

### Structural analysis of Silicon transporter protein

**Figure 2:**
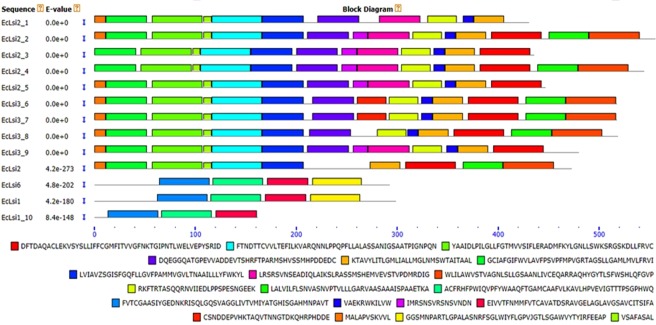
Organization of conserved motifs identified in finger millet silicon transporter proteins was predicted by MEME tools. The sequence and motifs identified were indicated by different coloured (1–20).

The nucleotide sequences were translated to amino acid sequences using Blastx tool available at NCBI (http:// blast.ncbi.nlm.nih.gov/ Blast.cgi). The basic information of physico-chemical properties including number of amino acids, molecular weight (kDa) and theoretical isoelectric point of silicon transporter (EcLsi) proteins was elucidated using Protparam tool of ExPASy (http://web.expasy.org/protparam/) (Table 1). The length of amino acid sequences of deduced silicon transporter proteins varied greatly ranging from 162 (EcLsi1_10) to 556 (EcLsi2_2) and molecular weight ranging from 17.04 (EcLsi1_10) to 60.26 kDa (EcLsi2_2) (Table 1). The highest and lowest theoretical iso-electric points (pI) of 8.52 and 5.22 were observed in EcLsi2 and EcLsi2_3 proteins respectively (Table 1). In addition, secondary structures of silicon transporter proteins were predicted by SOPMA tool and it was observed that the percentage of alpha helices (32.44 to 51.93), beta sheets (10.32 to 21.6), beta turns (3.25 to 5.56) and random coil (27.78 to 46.15) also varied greatly (Table 1). A total of six conserved motifs were identified in EcLsi2, while three conserved motifs each were identified in EcLsi1 and EcLsi6 proteins using the MEME search tool (http://meme.nbcr.net). The maximum of 15 motifs was identified in EcLsi2_2 and the minimum of 3 motifs in EcLsi1_10 protein. The unique motifs were also identified in finger millet silicon transporter proteins, EcLsi3_9 contained maximum two unique motifs i.e. motif-10 and −14, followed by EcLsi3_6 and EcLsi3_7which contained only one uniquemotif-17 (Fig. 2). But the most significant motifs possessing NPA/SPA domains, which is a feature of major intrinsic proteins, were found in 9 out of 13 silicon transporter proteins analyzed (Table 1). Particularly, NPA domain was identified in EcLsi2 and SPA domain in EcLsi1 and EcLsi6 proteins, indicating that NPA is highly conserved in finger millet Lsi2 proteins while SPA in Lsi1 and Lsi6 proteins. Further, the analysis of putative phosphorylation sites in silicon transporter proteins revealed that motifs 8 and 9 contained high number of conserved serine residues and were most probably the sites for phosphorylation (Table S4). The highest number of 22 potential serine phosphorylation sites was predicted in EcLsi2 (EcLsi2_2, EcLsi2_3, EcLsi2_4 and EcLsi2_5) and EcLsi3 (EcLsi3_9) proteins. The lowest number of 2 serine phosphorylation sites per gene were detected in EcLsi1 (EcLsi1_10) protein. EcLsi2 has an average of 18.17 potential serine phosphorylation sites per gene, which is considerably high compared to EcLsi1 and EcLsi3 proteins. Further, the sub-cellular localization of silicon transporter proteins was predicted using the Wolfpsort, CELLO2GO and TargetP servers (Table S5). The results predict that majority of silicon transporter proteins are located in the plasma membrane except EcLsi6 and EcLsi1_10 which are predicted to be located in endoplasmic reticulum and vacuole respectively. The amino acid sequences of silicon transporter proteins were used to build the tertiary structures by using Phyre2 tool. The 3-D structure of all silicon transporter protein revealed that all EcLsi proteins form the pore structure with helices, the typical characteristic of transporter proteins (Supplemental Fig. S1). Among them EcLsi2_1, EcLsi2_2 and EcLsi3_8 proteins contained maximum of 23 alpha helices each whereas the lowest of 7 alpha helices was observed in EcLsi1_10 protein. The transmembrane domain and topology of the silicon transporter protein was predicted by TMHMM2.0 and Protter tool respectively. All the identified silicon transporter proteins contained transmembrane domain indicating that all silicon transporter proteins are membrane bound and have an active role in transport across the cell. It was observed that EcLsi2 protein contained maximum number of 10 of transmembrane domains whereas EcLsi1_10 contained lowest 5 transmembrane domains (Supplemental Fig. S2). The topology prediction revealed that the EcLsi1 and EcLsi6 were the only proteins that had their N-terminal and C-terminal ends located in intracellular region (Supplemental Fig. S3), whereas, EcLsi2, EcLsi2_2, EcLsi2_4 and EcLsi3_9 showed extracellular location of N-terminal and C-terminal ends (Supplemental Fig. S3). The EcLsi2_1 was the only protein which showed intracellular N-terminal and extracellular C-terminal ends localization, while most of the silicon transporter protein showed extracellular N-terminal and intracellular C-terminal ends localization.

**Table 1:** Characteristics of deduced protein of Silicon transporter genes identified.

Gene Name	Deduced protein						Secondary Structure (%)				Number of Helices	TMHMM (TMHs)	Protter (TMHs)
	A.A. Length	MW (kDa)	pI	NPA/SPA Domain	Instability index	Stable or unstable	Alpha Helix	Beta Sheet	Beta Turns	Rando m Coil			
*EcLsi1*	299	32.15	7.09	NPA	34.96	Stable	32.44	17.06	4.35	46.15	11	6	6
*EcLsi2*	473	50.34	8.52	SPA	34.84	Stable	51.16	15.64	4.65	28.54	17	10	10
*EcLsi6*	293	31.34	6.64	NPA	39.10	Stable	37.20	18.77	3.41	40.61	12	6	6
*EcLsi2_1*	431	46.30	5.78	—	42.26	Unstable	46.87	13.46	3.25	36.43	23	5	7
*EcLsi2_2*	556	60.26	5.96	SPA	39.05	Stable	47.84	15.65	4.68	31.83	23	9	10
*EcLsi2_3*	436	47.64	5.22	—	43.02	Unstable	49.31	10.32	4.13	36.24	18	6	7
*EcLsi2_4*	545	59.19	5.80	SPA	39.72	Stable	51.93	14.13	4.77	29.17	19	9	10
*EcLsi2_5*	447	48.71	5.32	—	42.10	Unstable	48.77	11.86	4.47	34.90	19	6	7
*EcLsi3_6*	518	55.92	6.01	SPA	36.88	Stable	47.88	12.74	5.02	34.36	22	8	9
*EcLsi3_7*	518	55.92	6.01	SPA	36.88	Stable	47.88	12.74	5.02	34.36	22	8	9
*EcLsi3_8*	519	55.93	6.01	SPA	37.39	Stable	47.40	14.26	5.20	33.14	23	9	11
*EcLsi3_9*	480	52.05	5.24	—	39.45	Stable	46.46	14.79	3.54	35.21	18	7	8
*EcLsi1_10*	162	17.04	6.18	NPA	27.91	Stable	45.06	21.60	5.56	27.78	7	5	5

(A.A. – Amino Acid, MW- Molecular Weight, pI- Isoelectric point, kDa- Kilo Dalton, TMHs- Transmembrane Helices).

### Phylogenetic analysis of silicon transporter genes

**Figure 3:**
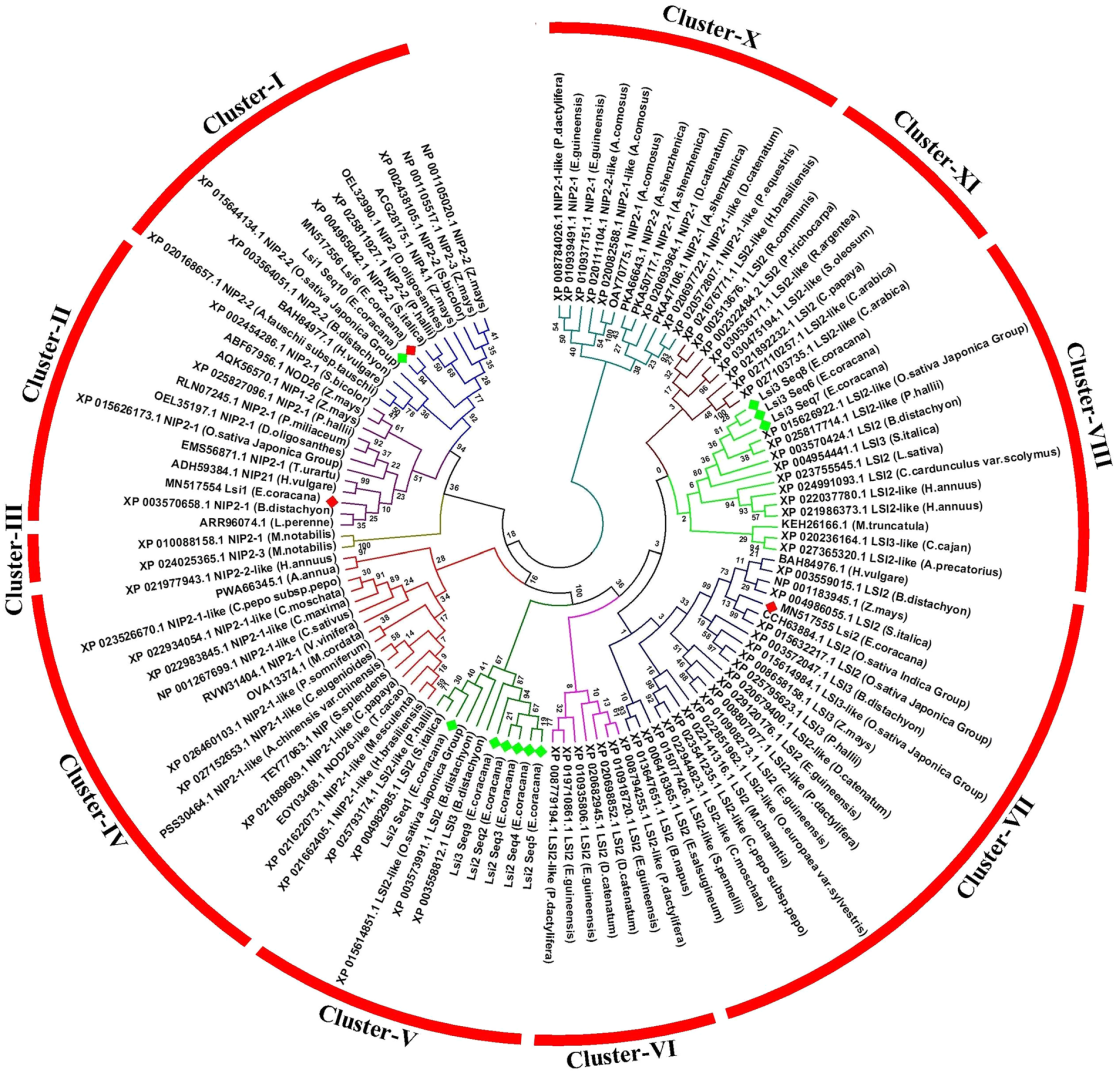
Phylogenetic tree of silicon transporter proteins identified from finger millet along with different plant species generated based on homology hit. Phylogenetic tree was constructed using the neighbor-joining algorithm with 1000 replications in MEGA7 software. The proteins from finger millet are indicated with the prefixes Ec. Numbers on the tree node indicates bootstrap values in per cent. The genes isolated from finger millet are presented in red color while the genes identified by transcriptome assembly are presented in green color.

The full-length deduced protein sequences of silicon transporter genes of *EcLsi1*, *EcLsi2* and *EcLsi6* isolated from Var. Suvra and their homologs obtained through transcriptome assembly along with NCBI BLASTX hit sequences were used for phylogenetic analysis based on MSA (Multiple Sequence Alignment). The phylogenetic tree was constructed by employing bootstrap test (1000 replications) and neighbor joining method. The phylogenetic analysis revealed that silicon transporter genes from different plant species were grouped into ten main clusters (cluster-I to cluster-X). Among them cluster-VII was the largest cluster comprising 21 genes mainly *Lsi2* including *EcLsi2* (MN517555) while cluster-III was the smallest cluster containing only 2 genes (NIP2-1 and NIP2-3) from *M. notabilis*. Cluster-V contained mainly *EcLsi2* genes whereas cluster-VIII contained *EcLsi3* genes. The genes *EcLsi1* and *EcLsi6* were confined to cluster-I along with NIP2s (Nodulin 26-like intrinsic protein), a *bona fide* silicon transporter (Fig. 3). *EcLsi1* and *EcLsi6*werefound to be closely related to *S. italica* followed by *P.hallii*, *Z. mays* and *S.bicolar* species with bootstrap value having 79 nodule stability (Fig. 3). It was observed that *Lsi1* and *Lsi6* are homologs belonging to Nod26-like major intrinsic protein (NIP2) subfamily of aquaporin protein family involved in silicon influx transport.*EcLsi2* was found to be closely related to S.*italica*, *B.distachyon* and *O.sativa* japonica group, and *EcLsi3* had more similarity with *O. sativa* japonica group and *P.hallii* (Fig. 3). The*Lsi2* and *Lsi3* efflux silicon transporters belong to anion transport family and have a significant identity with putative arsB transporter proteins. Furthermore, it was observed that *Lsi1* might have co-evolved with *Lsi6* genes in cereal species. Moreover, *EcLsi2*and *EcLsi3* might have evolved from *EcLsi1* and/or *EcLsi6* and/or from both genes due to the gene duplication (Fig. 3 and Supplemental Fig. S3).

### Expression analysis of silicon transporter genes

**Figure 4:**
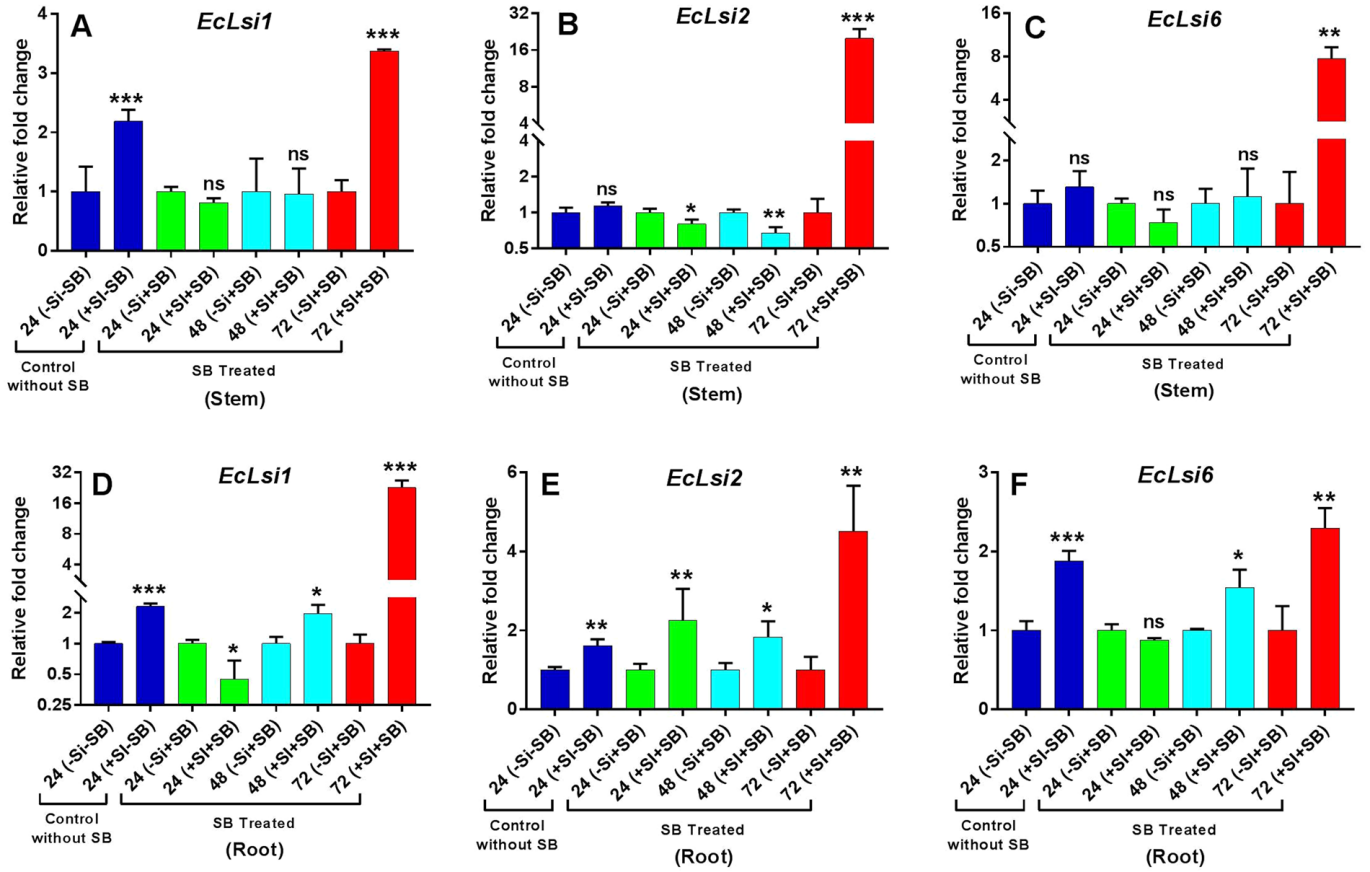
Expression of representative genes (*EcLsi1*, *EcLsi2* and *Eclsi6*) with (–Si–SB) control without Si and SB, and (–Si + SB) without silicon and with stem borer treatments at mentioned time points (24, 48 and 72 hpi) is considered as 1-fold after normalizing data with housekeeping gene (*EcActin*). The expression of genes in (+ Si–SB) control with Si without SB, and (+Si + SB) (with silicon and stem borer) is calculated as fold change. The fold change level of *EcLsi1*, EcLsi*2 and Eclsi6* are presented in fig (**A–C)** in stem and (**D—F**) in root of finger millet respectively. Error bars represents ± SD (*n* = 3). The star above the bars graph represents different level significance obtained from paired t-test (*) at 0.05, (**) at 0.01 and (***) at 0.001 alpha levels respectively.

The qRT-PCR analysis is was performed to understand the expression patterns of silicon transporter genes (*EcLsi1*, *EcLsi2* and *EcLsi6*) in both stem and root tissues in finger millet infested by stem borer and silicon amendment. The results showed that the expression levels of only. *EcLsi1* (P = 0.001) was significantly increased in stem tissue, whereas in roots, significant up-regulation of all three genes viz. *EcLsi1* (*P* = 0.000), *EcLsi2* (*P* = 0.004) and *EcLsi6* (*P* = 0.001) was observed in Si-treated plants (without SB infestation) over the non-silicon treated plants at 24 hpi (control) indicating that silicon alone was responsible for up-regulation of silicon transporter genes. Further, the expression analyses showed that all three tested genes (*EcLsi1*, *EcLsi2* and *EcLsi6)* were induced by stem borer infestation in both stem and root tissues as expected. Among the three tested silicon transporter genes, *EcLsi2* transcript had the highest expression observed in both stem and root tissues. However, the highest expression of *EcLsi2* was observed in non-silicon treated plants in roots and stems in silicon-treated plants at 24 hpi (hours of post infestation). In roots, the t-test analysis showed a significant up-regulation of *EcLsi2* (*P* = 0.057) and down regulation of *EcLsi1* (*P* = 0.018) transcripts in silicon-treated and stems borer infested plants at 24 h hpi. Moreover, a significant increase in *EcLsi1*, *EcLsi2* and *EcLsi6* was observed at 48 (*P* = 0.020, *P* = 0.029 and *P* = 0.013) and 72 (*P* = 0.000, *P* = 0.007 and *P* = 0.004) hpi respectively in silicon treated plants over the non-silicon treated plants. Among all three tested genes, the highest and significant up regulation of *EcLsi2* transcript was observed in root at 24hpi as compared to *EcLsi1* and *EcLsi6* in silicon treated plants. In stem, *EcLsi2* was the only silicon transporter gene significantly down regulated at 24 (*P *= 0. 0.035) and 48 (*P* = 0.005) hpi in silicon treated plants over the non-silicon treated plants. The only significant up regulation of all three tested genes viz. *EcLsi1*(*P* = 0.000), *EcLsi2* (*P* = 0.001) and *EcLsi6* (*P* = 0.002) was observed in stem at 72 hpi in silicon treated plants over the non-silicon treated plants. These results indicate that stem borer infestation alone was responsible for up- regulation of *EcLsi1* in root and *EcLsi2* in stem in non-silicon treated plants despite of silicon availability. A considerable depression was observed up to 72 hpi which might be due to the silicon starvation or because of differential expression related to silicon availability. This major regulation of silicon transporter genes might be a feedback control in plants according to the silicon status or saturation in plants. This result indicates that the up-regulation of silicon transporter genes at 72 hpi is crucial and might have the role in defense mechanism. Compared to non-silicon treated plants, the only significant increase in relative fold change was observed for *EcLsi2* transcript at all three time points viz. 24 (2.25), 48 (1.84) and 72 (4.51) hpi in root at 0.01, 0.5 and 0.01 level of significance respectively (Fig. 4E). The only significant decrease in fold change (0.45) was observed for *EcLsi1* at 24 hpi (*P* = 0.018) in root (Fig. 4D). However, a significant decrease in fold change for *EcLsi2* i.e. 0.80 and 0.68 was observed at 24 (*P* = 0.035) and 48 (*P* = 0.675) hpi in stem respectively (Fig. 4E). Moreover, the transcript levels of *EcLsi1* (1.97 and 22.57) and *EcLsi6* (1.54 and 2.29) were also significantly increased in root of silicon treated plants at 48 (*P* = 0.020 and *P* = 0.013) and 72 (*P* = 0.000 and *P* = 0.004) hpi respectively (Fig. 4D,F). In stem, a significant fold change for *EcLsi1*(3.38), *EcLsi2*(19.94) and *EcLsi6* (7.77) was observed only at 72 hpi in silicon treated plants (Fig. 4A–C).

### Expression analysis of defense hormone regulating genes

**Figure 5:**
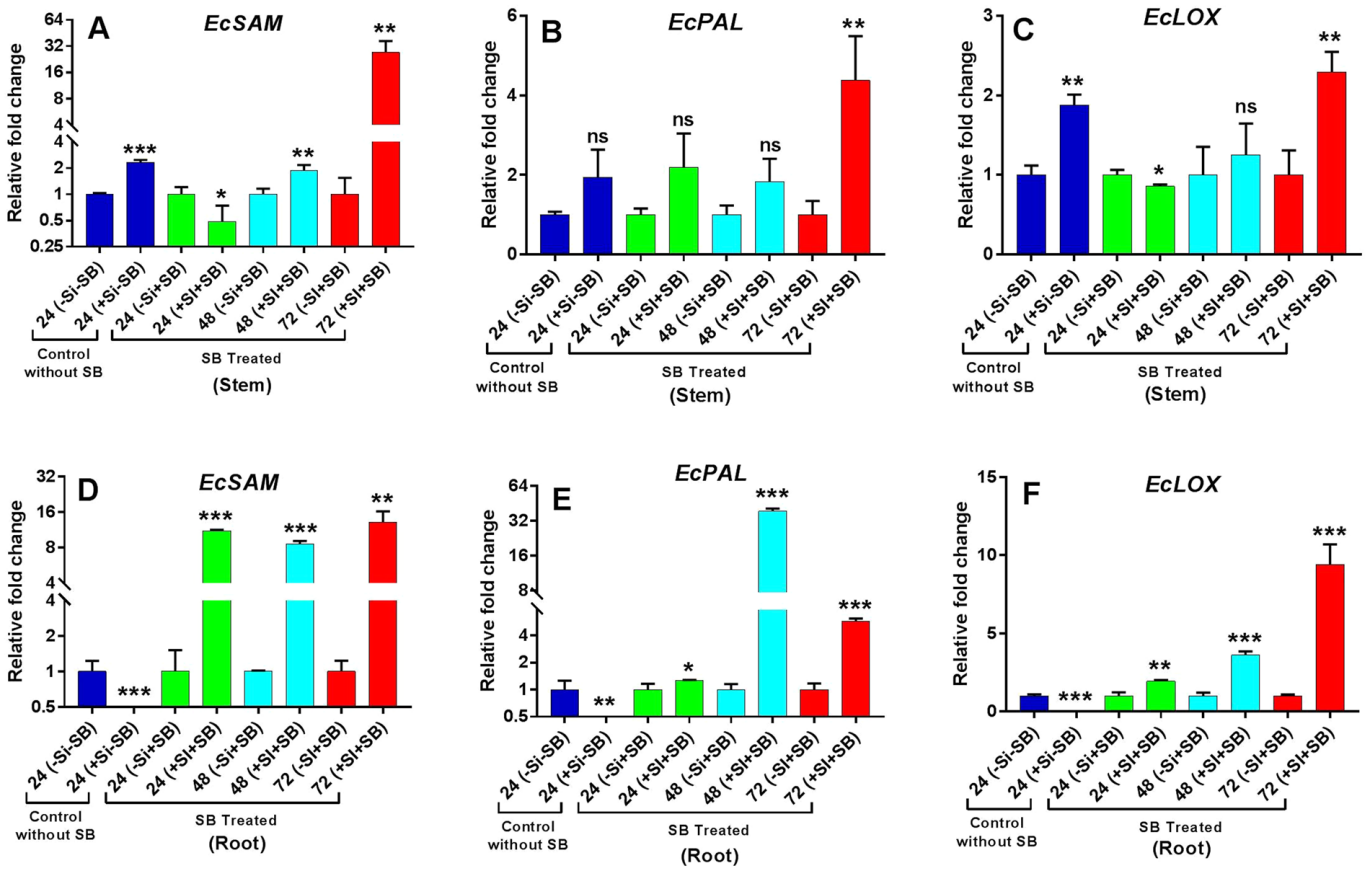
Expression of representative genes (*EcSAM*, *EcPAL* and *EcLOX*) with (–Si–SB) control without Si and SB, and (–Si + SB) without silicon and with stem borer treatments at mentioned time points (24, 48 and 72 hpi) is considered as 1 fold after normalizing data with housekeeping gene (*EcActin*). The expression of genes in (+Si–SB) control with Si without SB, (+Si + SB) with silicon and stem borer is calculated as fold change. The fold change level of *EcSAM*, *EcPAL* and *EcLOX *are presented in fig (**A–C**) in stem and **(D–F**) in root of finger millet respectively. Error bars represents ± SD (*n* = 3). The star above the bars graph represents different level significance obtained from paired t-test (*) at 0.05, (**) at 0.01 and (***) at 0.001 alpha levels respectively.

The relative expression of defense hormone regulation genes such as SAM (S-adenosyl-L- methionine), *PAL* (Phenyl-alanine ammonia-lyase) and *LOX* (Lipoxygenase) of Ethylene (ET), Salicylic acid (SA) and Jasmonic acid (JA) pathway respectively were analyzed. The expression levels of *EcSAM* (*P* = 0.000) and *EcLOX* (*P* = 0.000) genes were statistically significant at 24 hpi with mild transcriptional up-regulation in stem tissue with Si treatment (without SB infestation) indicating that the promoter(s) of these genes might be induced in response to silicon treatment, whereas down regulation of *EcSAM* (*P* = 0.001), *EcPAL* (*P* = 0.002) and *EcLOX* (*P* = 0.000) genes with no detectable transcripts were observed in root tissue. These observations indicate that these genes show spatial-temporal expression patterns. Further, the qRT-PCR analyses showed that all three tested genes were induced by stem borer infestation as expected. However, the highest significant increase of *EcSAM* (*P* = 0.053) and *EcLOX*(*P* = 0.022) was observed in non-silicon treated plants in stem at 24 hpi. T-test statistical analysis also showed that *EcSAM* and *EcPAL* transcripts were significantly increased in stem at 48 (*P* = 0.011 and 0.027) and 72 (*P* = 0.009 and 0.007) hpi in silicon treated plants over the non-silicon treated plants respectively. The up- regulation of *EcLOX* was found to be significant only at 72 hpi (0.004) over the non-silicon treated plants in stem. In case of roots, the stem borer infestation was not considerably responsible for induction of all three tested genes in non-silicon treated plants. But in the presence of silicon, the stem borer infestation was responsible for significantly increasing the transcript level of all three tested genes viz. *EcSAM*, *EcPAL* and *EcLOX* in roots at all three time periods i.e. 24 (*P* = 0.000, *P* = 0.049 and *P* = 0.002), 48 (*P* = 0.000, *P* = 0.000 and *P* = 0.000) and 72 (*P* = 0.002, *P* = 0.000 and *P* = 0.000) hpi over the non-silicon treated plants. In root, the highest transcript level was observed for *EcSAM* as compared to *EcPAL* and *EcLOX* at 24 hpi in silicon treated plants. Moreover, an antagonistic feedback control was observed in root between *EcPAL* (Salicylic Acid) and *EcLOX* (Jasmonic Acid) in silicon treated and stem borer infested plants. These results indicate that silicon was responsible for amplifying the transcripts level of all three tested genes in root under the stem borer infestation stress in finger millet plants. In stem, compared to non-silicon treated plants, significant fold change increases i.e. 27.04, 4.37 and 2.29 were observed for *EcSAM*, *EcPAL* and *EcLOX* transcripts at 72 hpi (*P* = 0.009, *P* = 0.007 and *P* = 0.004) respectively (Fig. 5A–C). In addition, *EcSAM* transcript was found to be increased by1.87 fold in stems at 48 hpi (*P* = 0.011) (Fig. 5A). Compared to non-silicon treated plants, decrease in transcript levels of *EcSAM* (0.48-fold change) and *EcLOX* (0.85-fold change) was seen in stems of silicon treated plants at 24 hpi (*P* = 0.053 and *P* = 0.022, respectively) (Fig. 5A–C). In case of root, the significant increase in expression was observed for all three tested genes at all-time points compared to non-silicon treated plants, the transcripts level of *EcSAM*, *EcPAL* and *EcLOX* were induced 11.11 (*P* = 0.000), 1.26 (*P* = 0.049) and 1.93 (*P* = 0.002) fold at 24 hpi respectively (Fig. 5D–F). At 48 hpi, the transcripts levels of *EcSAM*, *EcPAL* and *EcLOX* were increased by 8.53 (*P* = 0.000), 38.59 (*P* = 0.000) and 3.61 (*P* = 0.000) fold and at 72 hpi, they increased by 13.09 (*P* = 0.002), 5.69 (*P* = 0.000) and 9.41 (*P* = 0.000) fold respectively in root as compared to non-silicon treated plants after stem borer infestation (Fig. 5D–F).

### Inhibitory effect of Silicon content on tunnel length

**Figure 6:**
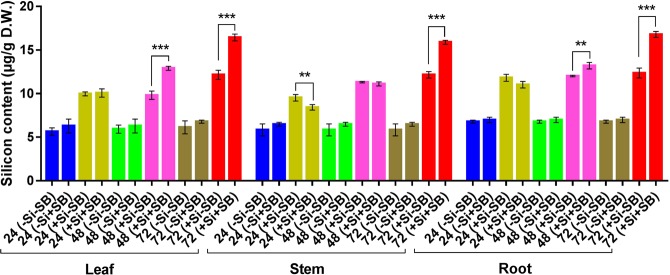
Silicon content of finger millet plant (µg/g) on dry weight basis in leaf, stem and roottissues at different time points. Error bars represents ± SD (n = 3). The star above the bars graph represents different level significance obtained from paired t-test (*) at 0.05, (**) at 0.01 and (***) at 0.001 alpha levels respectively.

Silicon amendment with silicic acid hydrate in nutrient solution (1 mM/L) increased the silicon concentration in finger millet plants (Fig. 6). Silicon amendment alone was responsible for increasing the Si content in leaf, stem and root of Si-treated plants significantly as compared to non-treated plants at all three time-points i.e. 24, 48 and 72 hpi. The silicon content of Si-treated and stem borer infested plants was significantly increased from 9.82 to 12.90 µg/g of dry weight (*P* = 0.000) in leaf and from 12.00 to 13.20 µg/g of dry weight (*P* = 0.005) in root over non silicon treated plants at 48 hpi. The silicon content decrease was observed in stem at 24 hpi (*P* = 0.018) in Si-treated plants over the non-silicon treated plants. In Si-treated plants, the silicon content increased significantly in all the three plant tissues i.e. leaf (*P* = 0.000), stem (*P* = 0.000) and root (*P* = 0.000) at 72 hpi as compared to non-silicon treated plants (Fig. 6). The maximum silicon content was observed in roots (16.78 µg/g), followed by leaves (16.43 µg/g) and stem (15.88 µg/g) at 72 hpi in Si-treated plants.

**Figure 7:**
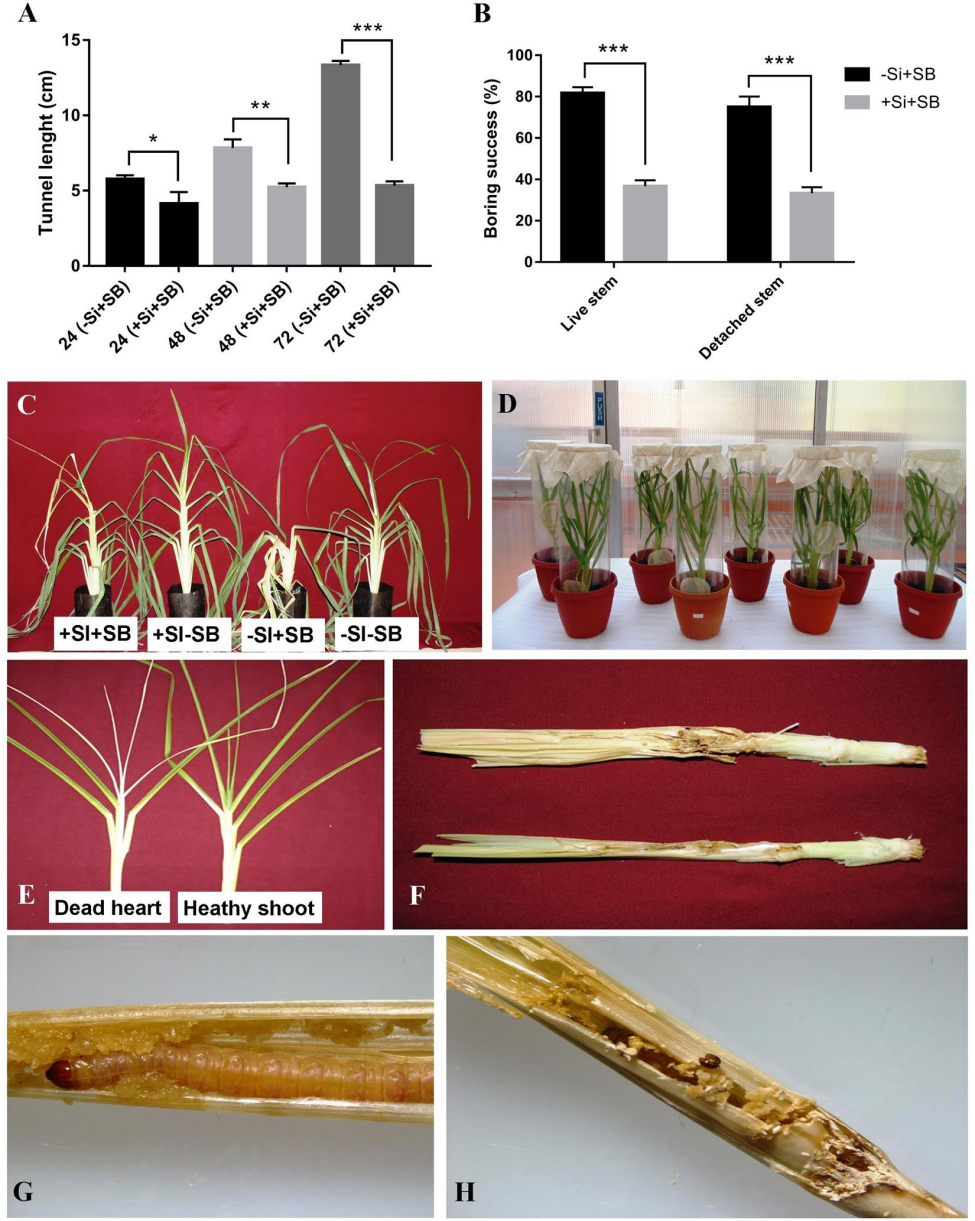
Silicon amendment reduces stem borer infestation in finger millet plants. (**A)**. Effect of silicon on feeding tunnel length (cm) measured with/without Si Treatment at different time points. (**B**). Larval boring success percentage of *S. inferens larvae* on Si-treated and un-treated plants of finger millet on live stem in greenhouse and on detached stem in laboratory. Error bars represents ± SD (n = 5). The star above the bars graph represents different level significance obtained from paired t-test (*) at 0.05, (**) at 0.01 and (***) at 0.001 alpha levels respectively. (**C)**. Development of dead heart symptom in finger millet plants after one week under different treatments. (**D**). Experimental set up for measuring tunnel length and boring percentage on live stem in response to Si treatment. (**E–H)**. Representative images of symptoms develop after SB infestation on finger millet plants (**E**) dead heart (**F**) feeding tunnel (**G**,**H**) frass.

An increase in silicon concentration in plant tissue particularly in stem significantly reduced the tunnel length in Si-treated plants over the non-silicon treated plants infested with stem borer at all three time-points i.e. at 24 hpi (*P* = 0.026), 48 hpi (*P* = 0.002) and 72 hpi (*P* = 0.000) (Fig. 7A). The highest tunnel length (13.33 cm) was recorded at 72 hpi in non-silicon treated plant and the lowest (5.23 cm) at 48 hpi followed by 5.33 cm at 72 hpi in Si-treated plants indicating that silicon accumulation inhibits the feeding and boring ability of stem borer in finger millet. Additionally, relative silicification of leaf sheath in response to Si amendment and SB infestation was also measured by SEM-EDX analysis (Supplemental Fig. S4 and S5). The polymerized silicon dioxide in leaf sheath form dumbbell shape-like structures which are sparsely distributed (Supplemental Fig. S5 A-1 to C-1). Silicon deposition was not observed in non-silicon treated plants of both SB infested and without SB infested plants (Supplemental Fig. S4). The SB infestation and Si addition led to intensive cell silicification in ragi leaf sheath at 72 hpi (Supplemental Fig. S5 D-2). In Si-treated plants, the highest silicon deposition (0.31 weight per cent) was observed in silicon amended and SB infested plants (+Si + SB) followed by 0.12 weight percent in non-silicon infested plants (+Si-SB) at 72 hpi. The lowest silicon deposition i.e. 0.10 and 0.06 weight percent was observed in + Si–SB and + Si + SB respectively.

### Boring success on live stem

The percentage of third instar larvae that bored into the live stem of ragi plants within 72 h varied significantly under greenhouse conditions in Si-treated plants (*P* = 0.000) (Fig. 7B,D). Overall, the maximum percentage of larvae that bored into the stem was higher in Si-treated plants (81.67 ± 1.67%) as compared to non-silicon treated plants (37.33 ± 1.45%). The results showed that silicon accumulation in silicon treated stem reduced the boring percentage by 45.71% as compared to non-silicon treated plants.

### Boring success on detached stem (‘detached stem’ assays)

Detached stem assays also showed the similar effects of Si on boring success of larvae (Fig. 7B,C,E). The results revealed that the percentage of boring success was significantly decreased in Si-treated plants (34.67 ± 2.03%) compared to non-silicon treated plants (75.67 ± 3.48%) (*P* = 0.000). It was observed that the success of boring percentage was reduced by 45.81% in Si- treated plants due to silicon accumulation compared to non-silicon treated plants.

## Discussion

The beneficial effects of silicon in suppressing the numerous biotic and abiotic stresses by inducing the plant resistance have been evident in a wide range of plant species^29^. The majority of silicon accumulating crops are from the cereal family. Rice is well-known for its silicon uptake ability and can accumulate up to 10% of silicon on a dry weight basis in the shoot^9^. This higher accumulation of Si in different plant tissues was reported to be directly involved in physical barrier mechanism and indirectly in inducing the chemical defense mechanism against insect pests^30^. There is recent evidence that Si induces the defense mechanism against stem borers of economically important crops like wheat, rice, maize and sugarcane^11^. This induced defense mechanism depends on the silicon accumulation ability of crop plants governed by silicon transporter genes and their differential expression during stress. More recently, genes involved in Si uptake and distribution (*Lsi1*, *Lsi2*, and *Lsi6*) were identified in numerous plant species^23,24^.

In the present investigation, an attempt has been made to amplify and isolate the silicon transporter genes viz. *EcLsi1*, *EcLsi2* and *EcLsi6* from finger millet as it is not reported earlier. We isolated the three silicon transporter genes from Suvra variety of finger millet and it was observed that PCR amplification of *EcLsi1*, *EcLsi2* and *EcLsi6 *produces 900 bp, 1422 bp and 882 bp long fragments respectively. After sequencing, the nucleotide sequences of all three genes were searched using BLAST tool in NCBI for identification and similarity search against different plant species. The BLAST results showed that all three isolated genes had similarity with silicon transporter genes. Recently, Sun *et al*.^31,32^ and Zellner *et al*.^33^ also isolated the silicon transporter genes from cucumber and tobacco plants. In addition, transcriptome library of finger millet was also assembled to nine homologs of silicon transporter genes. Total of ten expression transcripts of silicon transporter family were identified and named according to the BLAST hit identity such as *EcLsi1* (1), *EcLsi2* (5), and *EcLsi3* (4). Similarly, several homologous genes *OsLsi1*, *OsLsi2* and *OsLsi6* have been isolated and functionally characterized in barley^34,35^, maize^27,35^, wheat^36^ and horsetail^23^.

Multiple sequence alignment of silicon transporter proteins amino acid residues showed that EcLsi1 and EcLsi6 were highly homologous to each other and also shared homology with other plant Lsi1s and Lsi6s. EcLsi2 and EcLsi3 shared homology with each other as well as with other Lsi2s and Lsi3s sequences of different plant species. Specific characteristics of NPA domain motifs existed in EcLsi1 and EcLsi6 while EcLsi2 and EcLsi3 contained SPA domain motifs and four amino acid residues for an ar/R selectivity filter^36^. Deshmukh *et al*.^37^ reported that all Nodulin 26-like intrinsic proteins (NIP2s) are *bona fide* silicon transporters which include Lsi1 and Lsi6 and contain NPA domain as a characteristic feature. However, EcLsi2 and EcLsi3 lack NPA domain motifs but instead contain SPA domain motif which is the characteristic feature of uncharacterized intrinsic proteins (XIPs) as reported by Deshmukh *et al*.^37^. Further, the subcellular localization prediction revealed that all silicon transporter proteins were located in plasma membrane. This subcellular localization of characterized silicon transporters is consistent with the previous studies reported in other plant species^38^. Similarly, Sun *et al*.^32^ found that CsLsi2 was localized at the plasma membrane which is the same as for HvLsi2 and ZmLsi2. These results indicate that EcLsi1, EcLsi2, EcLsi3 and EcLsi6 may be involved in silicon transport across the plasma membrane. However, Wolfpsort tool predicted that EcLsi6 and EcLsi1_10 may be located in endoplasmic reticulum and vacuole respectively suggesting that EcLsi6 and EcLsi1_10 play different roles in Si uptake.

Phylogenetic tree analysis revealed the evolutionary relationship between the silicon transporter genes among the different plant species. All the silicon transporter genes were grouped into ten main clusters (Cluster-I to X). Among them, cluster-V contained mainly *EcLsi2* genes whereas cluster-VIII contained *EcLsi3* genes, but both the clusters contain efflux silicon transporter genes belonging to anion transport family indicating that *EcLsi2* and *EcLsi3* are homologous to each other and also have a significant identity with putative arsB transporter proteins. Ma *et al*.^26^ also reported that Lsi2 proteins having a significant identity with arsB prokaryotic arsenic transporters are involved in anion transport. Other two silicon transporter proteins, EcLsi1 and EcLsi6, were grouped together in cluster-I along with NIP2s (Nodulin 26-like intrinsic protein), *bona fide* silicon transporters. It was observed that *Lsi1* and *Lsi6* are homologs belonging to Nod26-like major intrinsic protein (NIP2) subfamily of aquaporins involved in silicon influx transport. Ma *et al*.^28^ reported that *OsLsi6* is the only homolog of *OsLsi1* in rice and belongs to NIP group. Moreover, *EcLsi1* and *EcLsi6* were found to be closely related with other millet family member *S.italica* and also with other grass family members like *P.hallii*, *Z.mays* and *S.bicolar*. *EcLsi2* was found to be closely related to *S. italica*, *B.distachyon* and *O.sativa* japonica group, while *EcLsi3* had more similarity with *O. sativa* japonica group and *P.hallii*. Furthermore, it was observed that *Lsi1* might have coevolved with *Lsi6* in cereal crop species. Moreover, *EcLsi2* and *EcLsi3* might have evolved from *EcLsi1* and/or *EcLsi6* and/or from both genes due to a duplication event during evolution. The phylogenetic analysis of silicon transporter genes was found to be useful to establish the evolutionary relatedness and divergence in different plant species^32,39^.

The transmembrane domain and topology of all silicon transporter protein was predicted to have multi-spam helices as majority of protein structure was made up of alpha helices. It was confirmed that silicon transporter proteins are membrane-bound and involved in silicon transport across the cell. Ma and Yamaji^9^ have reported that the *Lsi1* gene is predicted to encode a membrane protein similar to aquaporins, the water channels proteins. We also observed that both EcLsi1 and EcLsi6 proteins contained 6 transmembrane domains. Montpetit *et al*.^36^ demonstrated that silicon transporters have specific characteristics of six transmembrane domains which are well conserved in typical aquaporin proteins. However, EcLsi2 contained 10 transmembrane domains which might be due to duplication of EcLsi1 and EcLsi6 or fusion of both during evolution as evident by phylogenetic analysis. Likewise, Marron *et al*.^40^ suggested that the symmetrical 10 transmembrane domains of silicon transporter proteins (SIT) have independently evolved multiple times via duplication and fusion of 5 transmembrane domains of SIT-Ls, an extended family of related transporters present in some non-silicified organisms.

The present study revealed that the *EcLsi1*, *EcLsi2* and *EcLsi6 *were differentially expressed in root and stem tissues of finger millet if infested by stem borer in silicon treated and untreated plants. The qRT-PCR analyses confirmed that *EcLsi1* was primarily expressed in root followed by *EcLsi6* and *EcLsi1*, indicating that *EcLsi1*is mainly involved in silicon uptake in root along with *EcLsi6* and *EcLsi1* genes. However, Wangkaew *et al*.^41^ showed that the *Lsi1* and *Lsi2* genes are highly expressed in rice root and involved in Si uptake in all growth stages. Ma and Yamaji^38^ also confirmed that *Lsi1* and *Lsi2* genes are involved in silicon uptake in rice root by knocking out *Lsi1* and *Lsi2* genes, resulting in significant decrease in Si uptake in the rice roots. Our study indicates that *EcLsi1* is a highly expressed silicon efflux transporter involved in silicon uptake by root of finger millet, together with influx transporters, *EcLsi6* and *EcLsi1*, during the stem borer infestation. In case of stem, the highest expression was observed for *EcLsi1* followed by *EcLsi6*, indicating that *EcLsi1* is the major silicon efflux transporter in root as well as stem during stem borer infestation and *EcLsi6* is crucial in stems for silicon distribution in finger millet during stem borer attack. Similarly, Wangkaew *et al*.^41^ showed that the transcript level of *Lsi6* was highly expressed in node, internode and flag leaf, especially at booting stage, resulting in increase of silicon concentration in panicle of rice plants. This result is also consistent with the suggestion by Yamaji and Ma^42^ that *Lsi6* was involved in Si transportation to upper part of the rice plant. The expression of all three tested genes viz. *EcLsi1*, *EcLsi2* and *EcLsi6* were found to be significantly increased compared to non-silicon treated plants which indicates that the up-regulation of all these silicon transporter genes is crucial at 72 hpi against the stem borer attack. The results indicate that stem borer infestation alone was responsible for up-regulation of *EcLsi1* in root and stem in non-silicon treated plants regardless of silicon availability. Thereafter, a considerable depression was observed up to 72 hpi that might be due to the silicon starvation or by a differential expression to the silicon availability. This major regulation of silicon transporter genes might be a feedback control mechanism of plants according to the silicon status or saturation in plants. This result also indicates that the up-regulation of silicon transporter genes at 72 hpi is crucial and might have a role in defense mechanism. Ye *et al*.^43^ reported that the reduced steady-state transcript levels of the Si transporters *OsLsi1*, *OsLsi2*, and *OsLsi6* were observed in Si-pretreated plants after LF (Leaf Folder) attack. The common phyto-hormones salicylic acid (SA), jasmonic acid (JA) and ethylene (ET) play primary roles in orchestrating plant defense responses^15^. JA is suggested to regulate defenses against both cell-content-feeding and tissue-chewing insects^16,17^. In the present study, the relative expression of genes encoding *SAM* (S-adenosyl-L-methionine), *PAL* (Pheny-alanine ammonia-lyase) and *LOX* (Lipoxygenase) involved in ethylene (ET), Salicylic acid (SA) and Jasmonic acid (JA) pathways respectively were analyzed. The qRT-PCR analyses showed that all three tested genes were induced by stem borer infestation as expected. The transcript levels of *EcSAM* were significantly increased at 48 and 72 hpi in stem whereas in root they were increased at all-time points i.e. at 24, 48 and 72 hpi in silicon treated plants indicating that silicon hasa direct role in triggering the ethylene-dependent defense pathway in finger millet against stem borer. Previous studies also indicate that ethylene works synergistically with jasmonic acid in regulation of plant defense responses against herbivorous insects^44,45^. Further, the expression analyses revealed that the transcript levels of *EcPAL* (Salicylic acid) were significantly increased at 48 and 72 hpi whereas *EcLOX* (Jasmonic acid) transcript significantly decreased and increased in silicon treated plants in stem at 24 and 72 hpi respectively. This result indicates that stem borer infestation and silicon application might have induced synergistic JA- and SA-dependent defense mechanisms in finger millet. Kahl *et al*.^46^ stated that attackers may manipulate plants for their own benefit by shutting down induced defenses by influencing the signaling network. However, an antagonistic feedback control mechanism was observed in roots between *EcPAL* (Salicylic Acid) and *EcLOX* (Jasmonic Acid) in silicon treated and stem borer infested plants. A significant increase in transcript level of *EcPAL *and *EcLOX* was observed in root indicating that silicon was responsible for amplifying the JA and SA levels under the stem borer stress in finger millet plants. Spoel *et al*.^47^ demonstrated that the SA and JA is antagonistic to each other and simultaneously induced the resistance against chewing and piercing-sucking insects.

The study was conducted to assess the effect of Si on stem borer infestation through length of the tunnel and boring on live stem under greenhouse and on detached stem (cut stem assay) in the laboratory. The results revealed that Si amendment significantly increased the silicon accumulation at 48 hpi in leaf and root and at72 hpi, silicon concentration was increased in leaf, stem and root. This increase in silicon accumulation was negatively correlated with tunnel length and boring success percentage on both live and detached stems of Si-treated plants over the non-silicon treated plants. Bandong and Litsinger^48^ have observed that the rice stem hardening due to deposition of lignin and cellulose on leaf sheath by silica deposition caused less penetration and reduced feeding tunnel length. Tripathy and Rath^49^ also observed the reduced feeding tunnel length in rice in Si-treated plants over the control against yellow stem borer. SEM-EDX analysis also revealed that silicon deposition in Si-treated leaf sheath of SB infested plants at 72 hpi was up by0.31 weight per cent indicating that accumulation of silicon subsequently reduced the tunnel length and boring success percentage significantly compared to non-silicon treated SB infested plants. The reduction in feeding tunnel length and boring success might be due to the wearing of mandibles which would prevent further penetration of larvae in silicon treated plants. Silicon is also known to reduce the digestibility of feed in the insect diet and hence an increased in silicon uptake in Si-treated plants might have inhibited the larval digestion. Studies by Ranganathan *et al*.^50^ and Chandramani *et al*.^51^ also support the findings of the present investigation.

In conclusion, the present study identified the silicon transporter genes from finger millet. The results highlighted that *EcLsi1 *and *EcLsi6* genes are homologous silicon influx transporters while *EcLsi2* and *EcLsi3* genes are homologous silicon efflux transporters involved in uptake of silicon in finger millet. All silicon transporters proteins are membrane bound and predicted to be localized in plasma membrane allowing Si uptake in the finger millet plants. The phylogenetic and topology results suggest that *EcLsi1* and *EcLsi6* have co-evolved during evolution while *EcLsi2* and *EcLsi3* are evolved from either *EcLsi1* and/or *EcLsi6* or by fusion of both due to duplication event. The expression analyses indicate that stem borer infestation and silicon amendment increase the transcript levels of all three silicon transporter genes as well as defense phytohormone regulation genes significantly as compared to non-silicon treated plants in both stem and root at 72 hpi, indicating the crucial role of Si in inducing the defense mechanism against stem borer in finger millet. These findings also suggest that silicon transporters genes and defense phytohormone regulating genes act synergistically to mitigate the biotic stress induced by stem borer attack. The results also showed that Si amendment has a direct role in physical defense mechanism against the *S.inferens* due to intensified silicification in ragi leaf sheath which ultimately decreased feeding tunnel length and percent of boring success in ragi plants.

## Materials and Methods

### Plant materials and growth conditions

Finger millet seeds of var. Suvra susceptible to stem borer were collected from Pulses Research Centre (AICRP- All India Coordinated Research Project on Small Millets), OUAT, Berhampur, Odisha for the experiment. After germination, the germinated seeds were sown in plastic pots (27 × 19 × 4 cm) with soil less media (vermiculite 50% + Coco peat 50%) where the plants were supplemented with Hoagland solution^52^. After thirty days, a single seedling was transplanted to aplastic pot (50 × 40 × 15 cm) for further experimentation. The nutrient solution was prepared using deionized water (pH 6.0 ± 0.1). Si amendment (+Si) was carried out by adding silicic acid (Silicic Acid Hydrate, Himedia) to the nutrient solution at 1 mM Si/L and a control without addition of silicic acid (−Si) was included. The selected silicon concentration i.e. 1 mM Si/L was based on Nikolic *et al*.^53^ which suggested the silicon uptake rate was proportional to the solution silicon concentration in the range of 0–1 mM. At pH 6.0, nearly all the silicon is in the form of silicic acid^54^. The nutrient solution was replenished every five days. The plants were cultured in a greenhouse to prevent damage from rain and naturally occurring pests. Pesticides were not used throughout the experiment. Ragi plants at 60 days after transplanting (DAT) were used in the experiments.

### Stem borer infestation and plants sampling

Ragi pink stem borer (SB) larvae were collected from ragi fields at AICRP-Small millet center, Berhampur, Odisha. The larvae were raised on susceptible var. Suvra until pupa formation. The pupas were collected and incubated in plastic jar covered with muslin cloth for adult emergence under the control conditions in growth chamber. After emergence, the adults were confined to caged susceptible ragi plants in the growth chamber for oviposition. A stock culture of the newly hatched first instar was maintained on ragi plants without Si addition in a growth chamber at 28 ± 1 °C, 70 ± 5% relative humidity (RH) and a 16 h photoperiod until the larvae reached the third instar stage used for infestation. The plants were treated with four treatments; (1) with Si addition and SB infestation (+Si + SB), (2) without Si addition and with SB infestation (−Si + SB), (3) with Si addition and without SB infestation (+Si-SB), and (4) without Si addition and SB infestation (-Si-SB)with three replications each (n = 3). The stems and roots were sampled at 24, 48, and 72 h post-infestation (hpi) time points and immediately frozen into liquid nitrogen and stored at −80 °C. The stem and root samples collected were used for expression pattern analysis of silicon transporter genes (*EcLsi1*, *EcLsi2* and *EcLsi6*) and defense related phytohormones (ET- ethylene, JA- jasmonic acid and SA- salicylic acid) regulating genes (*EcSAM*- S-adenosyl-L-methionine, *EcPAL*- Phenylalanine ammonia-lyase and *EcLOX*-lipoxygenase respectively). The leaf samples were also collected for cloning of silicon transporter genes (*EcLsi1*, *EcLsi2* and *EcLsi6*).

### Measurement of Si content

Three plants per treatments were uprooted for collection of leaves, stems and root separately. Three samples per treatment were collected at different time points such as 24 h, 48 h and 72 h and oven dried at 70 °C for 3 days. Oven dried samples were fine-grinded and sieved through a 60 mm sieve. All the samples were further dried at 60 °C for 48 h to remove the moisture. Si content was determined by using molybdenum blue calorimetry method with slight modifications^55^. The absorbance was measured in Lambda 365 UV/Vis Spectrophotometer (PerkinElmer, USA) at 650 nm. The standard curved was drawn from Silicon standard solution provided by Merck Millipore (Germany). Subsequently, the Si content was determined from linear regression equation: y = 0.0067 × – 0.0121, R = 0.9977 and expressed as µg/g silicon in dry weight. In addition, scanning electron microscopy and energy dispersive X-ray analysis was performed to determine the relative Si content and silica deposition on leaf sheath surface of Si-treated and non-treated infested and non-infested plants coupled with EDX microanalysis mapping. EDX spectra of silicon were obtained using a sensitive mode with the scanned time of 3 min. The identification of the elements was done using auto identification against the built-in elemental database library.

### Larval boring success study on live stem (in greenhouse)

Intact plants grown in the greenhouse was tested for larval boring success study. An additional set was also taken with Si-treated and non-treated greenhouse grown plants to assess the boring success of larvae. The no choice-study was performed by confining the third instar larvae to either Si treated or untreated control plants. The larval boring success was determined as the percentage of third instar larvae entering the stems within 72 h of being placed on plants. An experiment was set up in randomized block design (RBD) with five replications. After thirty days, the plants were infested with five third instar larvae of *S. inferens* per plant (Fig. 7D). The percentage of larvae bored into the stem was calculated by counting the larvae that remained outside the stems after 72 h of confining the insects with the plants. The larval boring was confirmed by observing the visible entry holes into the stem and the debris and fecal matter coming out of the stem. Based on these data, the boring success was calculated by using the following formula:

$${\rm{Boring}}\,{\rm{success}}=\frac{{\rm{Number}}\,{\rm{of}}\,{\rm{larvae}}\,{\rm{bored}}\,{\rm{into}}\,{\rm{the}}\,{\rm{stem}}}{{\rm{Total}}\,{\rm{number}}\,{\rm{of}}\,{\rm{larvae}}\,{\rm{released}}\,{\rm{on}}\,{\rm{plant}}}\times 100$$

### Larval boring success study on detached stem (In Laboratory)

A detached stem study was also performed in the laboratory to assess the effect of Si on boring success of *S.inferens* on stem. Thirty days old greenhouse grown plants treated with silicon and without silicon were sacrificed and stems were collected for the experiment. The detached stems were prepared by cutting a 20 cm long piece from the base of the tiller and placed in glass test tubes (2.5 × 20 cm). The detached stem end facing the test tube base was sealed with parafilm and the other end was sealed using a foam plug. The freshness of the stem was maintained by keeping a wet cotton plug on open end of test tube (Fig. 7G-H). The experimental design was RBD with five replications. The treatments consisted of two test tubes i.e. Si-treated and non-treated (control) and infested with five third instar *S. inferens* larvae per test tube. The percentage of larvae bored into the stem was calculated by counting the larvae that remained outside the stems after 72 h of confining the insects with the plants. The larval boring was confirmed by observing the visible entry holes into the stem and the frass coming out of the stem. Based on these data, the boring success was determined.

### Isolation of Si transporter genes

Total RNA was isolated from stem, root and leaf tissues of finger millet variety “Suvra” amended with silicon under stem borer infestation at different time points by RNeasy Plant Mini Kit (cat. nos. 74903) as per the manual instructions. The concentration of RNA was quantified using the nanodrop spectrophotometer (Thermo scientific, USA). The absorbance and concentration were measured at 260 and 280 nm wavelength and the ratio of A260/A280 equal to 2.0 was considered as purified RNA. The total RNA was used for cDNA synthesis using Verso cDNA Synthesis Kit (Thermo Scientific, cat. nos. AB1453A) as per the manual instructions. Total RNA concentration of all samples was adjusted to 2 µg/µl. The PCR amplification of *EcLsi1, EcLsi2* and *EcLsi6 genes* was done by taking cDNA as template and gene-specific primers (Table S1). PCR amplification was carried out in 25 μL reaction volume containing template 25–50 ng of cDNA, 2.5 μl PCR assay buffer with 2.5 mM MgCl_2_ (Promega), 2.0 μl of 200 μM dNTPs, 1 μl of 1U of high fidelity Pfu polymerase (M/S Emerk Bioscience, India), 1 μl of 10 pmol of each forward and reverse primers and final volume was adjusted with nuclease free water (Hi-media, India). PCR amplification was carried out in Thermal Cycler (BioRad) with PCR cycling conditions as follows: initial denaturation at 94 °C for 4 min followed by 35 cycles of denaturation at 94 °C for 1 min, annealing at 56–60 °C for 50 s, elongation at 72 °C for 50 s and final elongation at 72 °C for 8 min. Tris–borate–EDTA (TBE) buffer was used for electrophoresis of amplified PCR product in 1.5% (w/v) agarose gel (M/S EMerck Bioscience,India) and the gels were photographed using gel documentation system (BioRad). The single bands from agarose gel were eluted using Quick PCR purification kit (Genei^TM^) as per the manual instructions. The purified single bands were sequenced using Sanger Dideoxy Sequencing method using 96 capillary high throughput sequencers (SaiGenom Labs Private Ltd, Cochin, India). The coding domain sequences (CDS) of *EcLsi1*, *EcLsi2* and *EcLsi6* genes isolated from finger millet variety Suvra has been submitted to GenBank, NCBI with the accession no. MN517554, MN517555 and MN517556 respectively.

### *De-novo* transcriptome assembly

The silicon transporter genes were mined from assembled transcriptome library of finger millet Vr. ML-365. The high throughput ILLUMINA (Next Seq 500) sequence reads of leaf tissue (Ac. No. SRR4021829) was retrieved from NCBI database^56^. The sequence quality of the raw reads and trimmed reads including GC content of the sequences were checked before assembly by Fast QC program (http://www.bioinformatics.babraham.ac.uk/Projects/fastqc/). Further the Trimmomatic program was used for cleaning the raw reads and quality control (http://www.usadellab.org/cms/?page=trimmomati)^57^. The parameters of trimming were adjusted to remove low quality pair reads (score ≤ 20), adaptor contamination and short reads (minimum length: 20 nucleotides). The cleaned reads were then assembled using Trinity assembler (https://github.com/trinityrnaseq/trinityrnaseq/wiki) with default parameters^58^. All the reads derived from the trinity assembly were further used to generate one combined assembly to obtain the longer and complete sequences using the Cap3 tool (http://seq.cs.iastate.edu/cap3.html). The redundant reads from the assembly were removed using the CD-HIT-EST tool (http://www.bioinformatics.org/cd-hit/). The non-redundant sequences were annotated by comparing with all possible silicon transporter proteins available at NCBI database using BLASTx search with cut-off  E-value ≤ 1.0e^-5^ and similarity score ≥ 60%. In addition, the reads without annotation result were separately compared with PLAZA 3.0, Uniprot and Ensemble plant protein database. This further survey of databases was to ensure the maximum annotation of putative contigs involved in silicon transport.

### *In-silico* sequence analysis

After sequencing, the nucleotide sequences of *EcLsi1*, *EcLsi2* and *EcLsi6* were edited in BioEdit software (http://www.mbio.ncsu.edu/BioEdit/bioedit.html) and further translated into deduced amino acid sequence by using the BLASTx tool available at NCBI (http:// blast.ncbi.nlm.nih.gov/Blast.cgi). Along with these three deduced amino acid sequences, other putative amino acid sequences obtained through transcriptome assembly were also included in sequence analysis. The physio-chemical properties including number of amino acids, molecular weight (kDa), theoretical isoelectric point and instability index of silicon transporter proteins were elucidated in Protparam tool of ExPASy^59^(http://web.expasy.org/protparam/) (Table 1). The amino acid sequences of silicon transporter genes were then subjected to homology modeling search to build the protein structure by using online Phyre2 tool with default parameters^60^. The 3-D structures of silicon transporter protein were visualized in PyMOL software. Further the sequences were subjected to Multiple Sequence Alignment (MSA) using ClustalX program using default parameters. The MSA file was further used in MEGA7 (Molecular Evolutionary genetic analysis) software for phylogenetic analysis (version 3.1; http://www.megasoftware.net)^61^. The phylogenetic tree was constructed using the neighbor-joining method with bootstrap test and poisson model. Separate phylogenetic tree was constructed for three genes and combined tree was also generated to establish the evolutionary relationship between silicon transporter genes and crop species.

### Expression analysis of genes

The relative expression pattern of silicon transporter genes viz. *EcLsi1*, *EcLsi2*, and *EcLsi6* and defense hormone regulating genes viz. *SAM (*Ethylene), *LOX (*Salicylic acid), and *PAL* (Jasmonic acid) were analyzed using qPCR (CFX96TM Real-Time System, Bio-Rad, USA) using SSO Fast Eva Green Supermixes (BioRad, USA) following manufacturer’s instruction. The housekeeping gene, *EcActin*, was used as an internal control for normalization of data obtained. Reaction conditions for thermal cycling were 95 °C for 60 s, followed by 40 cycles of 95 °C for 20 s, 60 °C for 15 s, then 72 °C for 30 s. Fluorescence data were collected during the cycle at 60 °C. The melting curve was used as an internal check for assessing quality of the gene amplification. The experiments were repeated twice independently with three replicates each time. The threshold cycles (Ct) were used to find the relative expression level of all the genes over control using the comparative 2^−ΔΔCt^ method^62^. Gene specific primers used in this study are listed in Table S6 and S7.

## Supplementary information

Supplementary information.
