# Supplementary material for: Silicon amendment induces synergistic plant defense mechanism against pink stem borer (Sesamia inferens Walker.) in finger millet (Eleusine coracana Gaertn.)
Source: Sci Rep. 2020 Mar 6;10:4229. doi: 10.1038/s41598-020-61182-0 (PMC7060215; doi:10.1038/s41598-020-61182-0)
Supplement: Supplementary file 1 — Supplementary information. [file 41598_2020_61182_MOESM1_ESM.pdf]

Silicon amendment induces synergistic plant defense mechanism against pink stem borer (*Sesamia inferens* Walker.) in finger millet (*Eleusine coracana* Gaertn.)

Kundansing Rajpalsing Jadhao[1*], Anuradha Bansal[2**] and Gyana R. Rout[1***+]

[1] Department of Agricultural Biotechnology, College of Agriculture, Odisha University of Agriculture & Technology, Bhubaneswar-751003, Odisha, India.
[2] Biointeractions and Crop Protection, Rothamsted Research, West Common, Harpenden, Hertfordshire, AL5 2JQ, United Kingdom
[1*]kjadhao1987@gmail.com/***grrout@rediffmail.com, [2**]anu.bansal@rothamsted.ac.uk
[+]corresponding author

Supplementary Figures

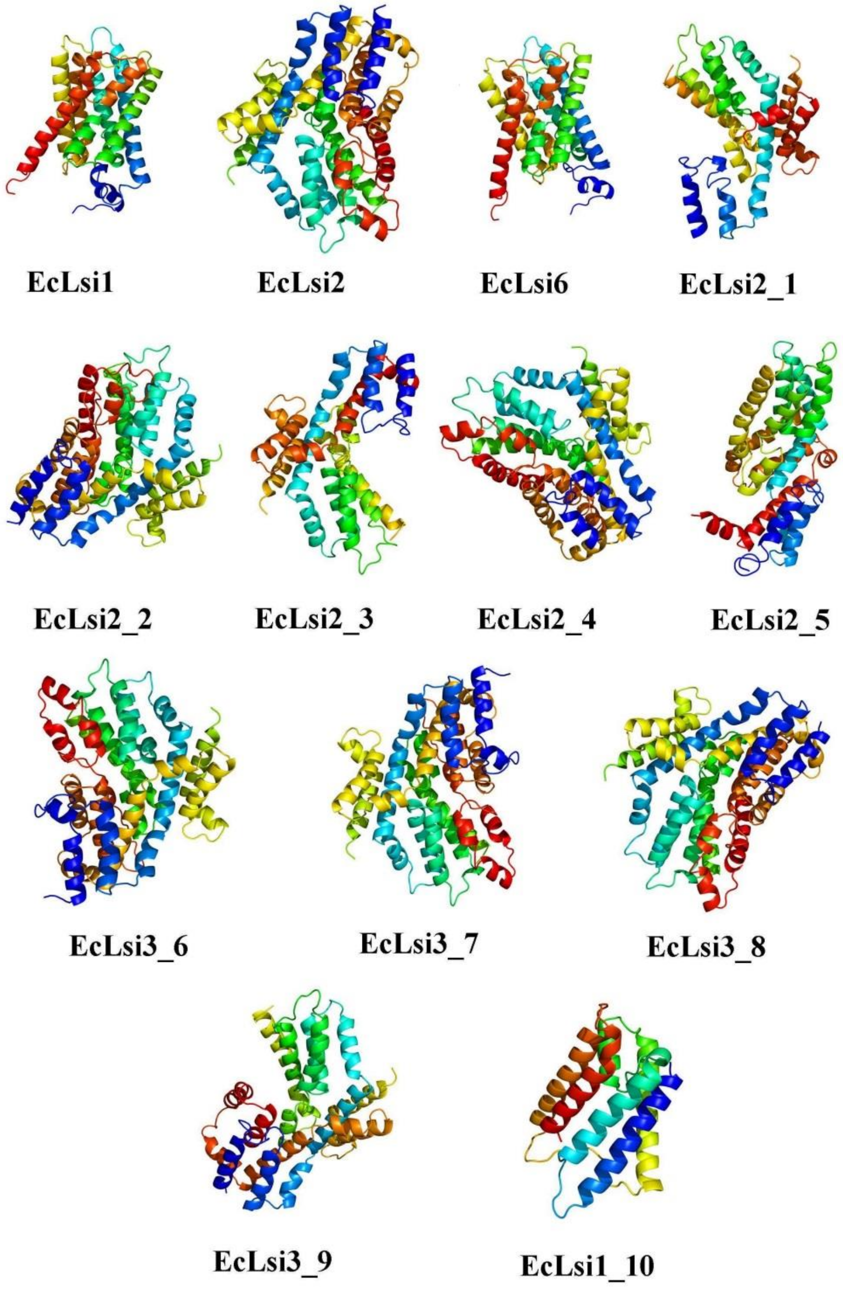

Fig S1. Tertiary (3D) protein structure of finger millet silicon transporter proteins depicted by Phyre2 server based on homology modeling. Models were visualized by rainbow color from N to C terminus.

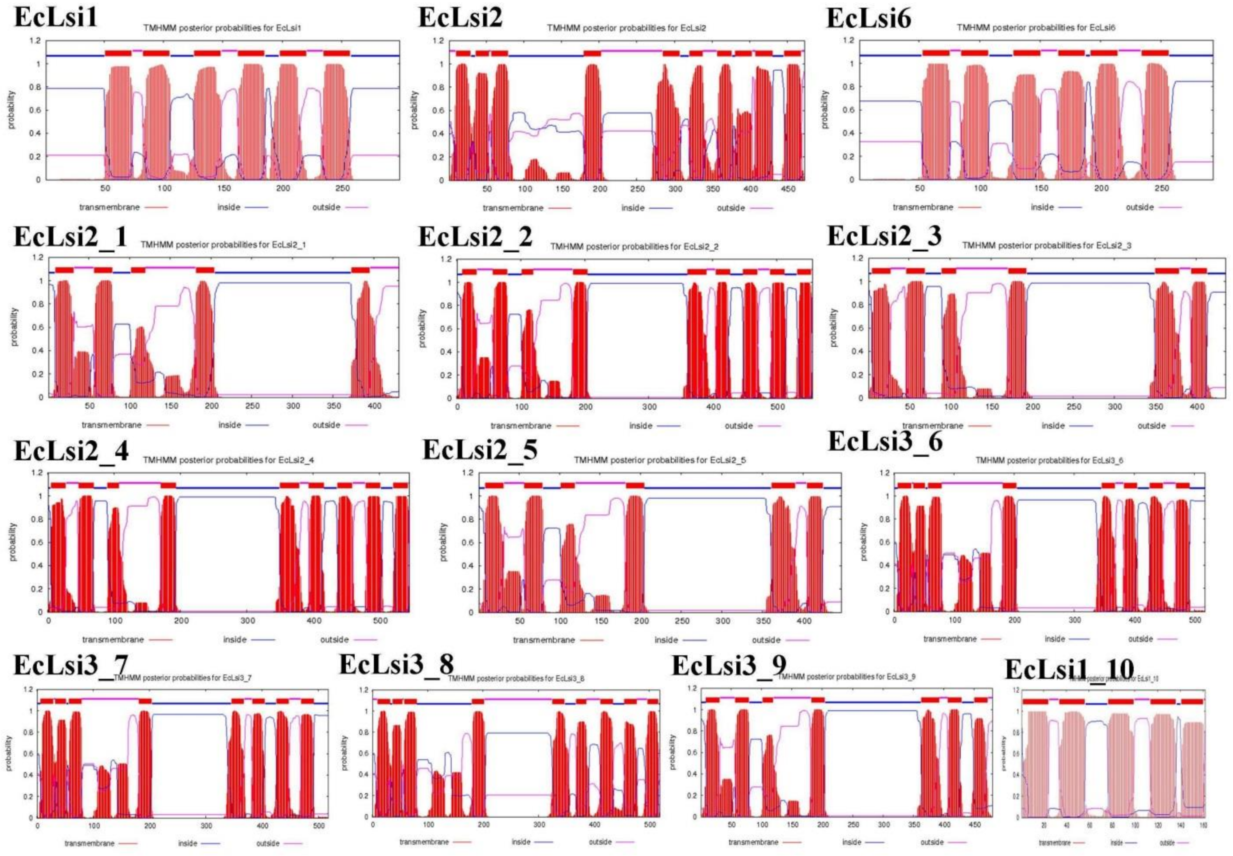

Fig S2. Transmembrane domains in finger millet silicon transporter proteins identified using TMHMM server.

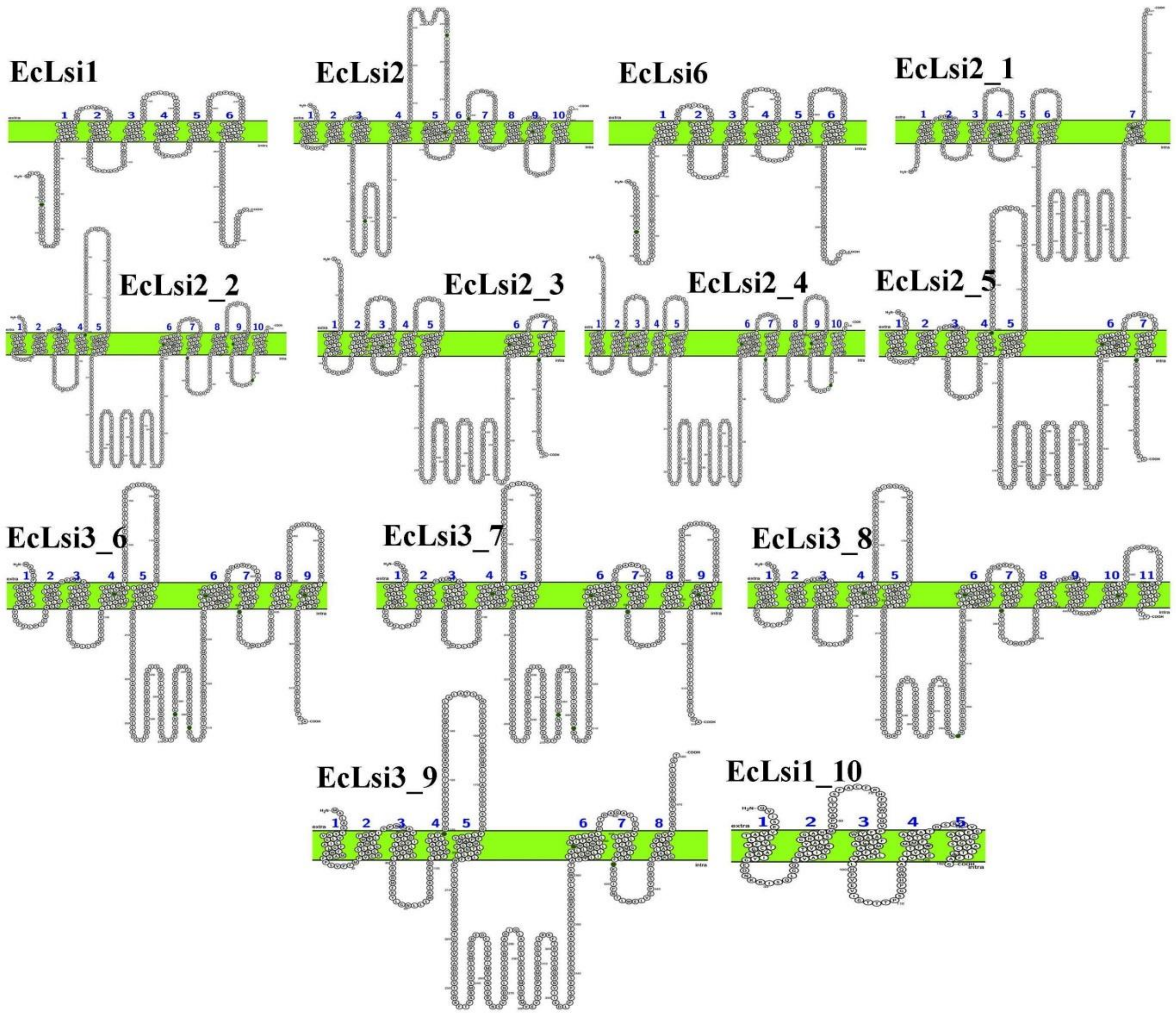

Fig S3. Topology of transmembrane domains of finger millet silicon transporter proteins depicted using Protter server.

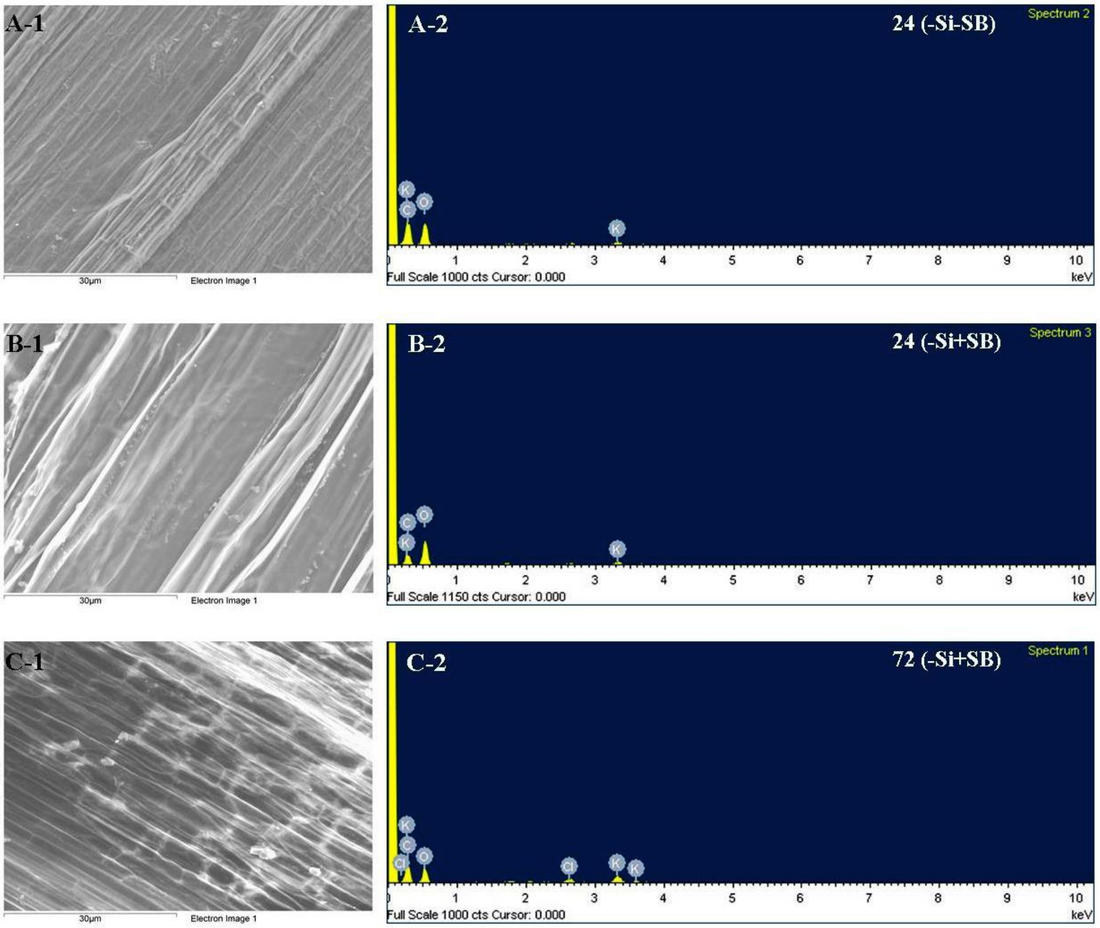

Fig S4. Scanning electron microscope image (250x, Bar = 30µm) **(A-1 to C-1)** and energy dispersive X-ray (EDX) **(B-2 to C-2)**. Silica cell mapping of ragi leaf sheath surface of "Suvra" variety at 24 and 72 hpi without Si addition (control).

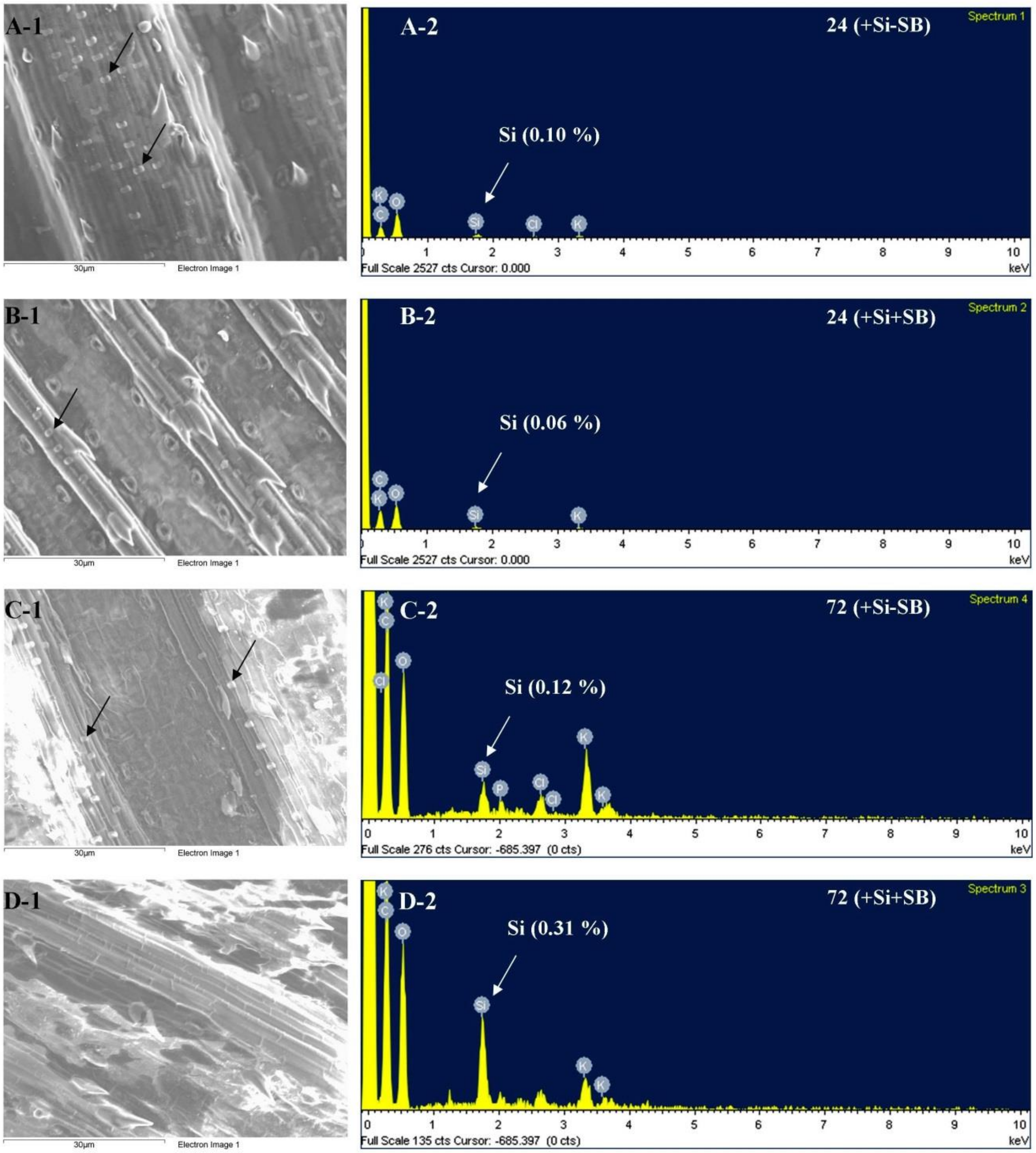

Fig S5. Scanning electron microscope image (250x, Bar = 30μm) **(A-1 to D-1)** and energy dispersive X-ray (EDX) (**A-2 to D-2**). Black arrow indicates the silica cell (dumbbell shape). White arrow denotes percent silicon mapped on ragi leaf sheath surface of “Suvra” variety at 24 and 72 hpi in Si-treated plants. (A-1 and 2): 24 hpi, with silicon and without SB infestation (+Si-SB). (B-1 and 2): 24 hpi, with silicon and SB infestation (+Si+SB). (C-1 and 2): 72 hpi, with silicon and without SB infestation (+Si-SB). (D-1 and 2): 72 hpi, with silicon and SB infestation (+Si+SB). Values in percent are means ± SD (n=3).

Supplementary Tables

Table S1. The sequences of silicon transporter genes specific primers designed from conserved sequences and their characteristics predicted by Primer 3 tool.

Primer Code	Based on conserved &Degenerate sequence primers (5'-3')	Length	Tm	GC%	Self	Self3	Primer site	Product length	Total CDS length
Lsi1-F1	TCGAGATCGAACTCCAGGGC	20	61.38	60.00	8.00	2.00	start 12	893 bps	913 bps
Lsi1-R1	CAGACGGGGATGTGGTCGAG	20	62.28	65.00	4.00	2.00	End 912		
Lsi1-F2	TCGAGGTCGAACTCCAGGG	19	60.68	63.16	8.00	2.00	Start 12		
Lsi1-R2	CACACTTGGATGTGGTCGAG	20	58.57	55.00	4.00	2.00	End 912		
Lsi2-F1	GCTCGCGTCTCTCCCCAAGG	20	65.00	70.00	4.00	2.00	Start 9	1412 bps	1473 bps
Lsi2-F1	GCTCTTGCTTCTGTGGCCAAGG	22	64.21	59.09	6.00	4.00	Start 7		
Lsi2-R1	TTCCGATGAGGGGGATGCCG	20	64.20	65.00	4.00	2.00	End 1463		
Lsi6-F1	GAACTACTCGAACGAGATCC	20	55.16	50.00	6.00	2.00	Start 45	806 bps	912 bps
Lsi6-R1	GGAGCTTGAAGGAGGAGAG	19	56.53	57.89	5.00	0.00	End 856		

Table S2. Completeness results of queried genes assemble by BUSCO tool

**Total number of core genes queried**	1440
**Number of core genes detected**	
**Complete**	1117 (77.57%)
**Complete + Partial**	1272 (88.33%)
**Number of missing core genes**	168 (11.67%)
**Average number of orthologs per core genes**	1.67
**% of detected core genes that have more than 1 ortholog**	48.61
**Scores in BUSCO format**	C:77.6%[S:39.9%,D:37.7%],F:10.8%,M:11.6%

Table S3. List of variant silicon transporter genes identified after transcriptome assembly based homology by BLSTX search tool.

Trinity contigs	NCBI Genes	Similarity %	E-value	Gene Name
Contig1692	XP_025793174.1	83.409	0	Silicon efflux transporter (EcLsi2_1)
Contig4561	XP_004985708.1	94.255	0	Silicon efflux transporter (EcLsi2_2)
Contig4562	XP_004985708.1	93.593	0	Silicon efflux transporter (EcLsi2_3)
Contig4563	XP_004985708.1	94.139	0	Silicon efflux transporter (EcLsi2_4)
Contig4564	XP_004985708.1	93.75	0	Silicon efflux transporter (EcLsi2_5)
TRINITY_DN41810_c0_g2_i2	XP_025817714.1	81.308	0	Silicon efflux transporter (EcLsi3_6)
TRINITY_DN41810_c0_g2_i3	XP_025817714.1	81.308	0	Silicon efflux transporter (EcLsi3_7)
TRINITY_DN41810_c0_g2_i5	XP_025817714.1	81.215	0	Silicon efflux transporter (EcLsi3_8)
TRINITY_DN44756_c0_g1_i1	XP_025791399.1	92.917	0	Silicon efflux transporter (EcLsi3_9)
TRINITY_DN41741_c0_g1_i7	BAG54792.1	93.21	1E-105	NOD26-like major intrinsic protein (EcLsi1_10)

Table S4. Details of serine phosphorylation sites detected in finger millet silicon transporter proteins. The Z score was estimated by an artificial neural network method phosphorylation prediction using NetPhos 2.0 Server (http://www.cbs.dtu.dk)

**Gene_ID**	**Protein Size**	**Phosphorylation sites**	**Serine Position**	**Sequence**	**Z score**
EcLsi1	299	9	5	MSTNSRTNS	0.778
			9	SRTNSRANF	0.660
			29	QNSGSMSSL	0.709
			72	AAAISASDQ	0.985
			80	QTRISQLGQ	0.978
			217	VSGGSMNPA	0.517
			270	NKDGSQKLS	0.965
			275	QKLSSFKLR	0.942
			283	RRLQSQSVA	0.717
EcLsi2	473	6	53	FHVISPDDA	0.992
			234	GVMKSPINN	0.970
			246	TDCDSPSMM	0.964
			255	TENISTKHP	0.867
			355	MAPYSKVNS	0.557
			359	SKVNSVGGI	0.738
EcLsi6	293	9	5	MAASSTSRT	0.694
			7	ASSTSRTNS	0.985
			11	SRTNSRVNY	0.988
			16	RVNYSNEIH	0.820
			29	VQSGSVVPT	0.747
			72	CGAASIYGE	0.971
			82	NKRISQLGQ	0.992
			219	VSGGSMNPA	0.539
			274	QKLSSFKLR	0.864
EcLsi2_1	431	15	241	DEVASHRFT	0.988
			250	PARMSHASS	0.991
			254	SHASSFNGG	0.676
			269	PMRRSDSMN	0.960
			271	RRSDSMNSR	0.971
			282	VDTMSMRSR	0.791
			285	MSMRSRSYN	0.810
			287	MRSRSYNSE	0.976
			290	RSYNSEGDI	0.994
			304	SMRASTMSQ	0.995
			307	ASTMSQEMV	0.985
			314	MVEVSTVSD	0.522
			317	VSTVSDRRD	0.993
			338	TRTTSHQRS	0.994
			342	SHQRSVIIE	0.973
EcLsi2_2	556	22	53	FRVISPEDA	0.997
			99	KSRGSKDLL	0.690
			208	WKYLSVEKD	0.848
			231	DEVTSHRFT	0.992
			240	PARMSHVSS	0.990
			243	MSHVSSMHP	0.677

			244	SHVSSMHPD	0.954
			262	MRSNSVRSN	0.996
			265	NSVRSNSVN	0.943
			267	VRSNSVNDN	0.997
			276	LRSRSVNSE	0.851
			279	RSVNSEADI	0.976
			293	SLRASSMSH	0.994
			294	LRASSMSHE	0.982
			296	ASSMSHEMV	0.993
			314	DIGGSSRKF	0.654
			315	IGGSSRKFT	0.993
			323	TRTASQQRN	0.975
			336	DLPPSPESN	0.995
			339	PSPESNGEE	0.991
			440	VEPYSRIDS	0.988
			444	SRIDSAKGV	0.972
EcLsi2_3	436	22	42	FRVISPEDA	0.997
			88	KSRGSKDLL	0.690
			197	WKYLSVEKD	0.848
			220	DEVTSHRFT	0.992
			229	PARMSHVSS	0.990
			232	MSHVSSMHP	0.677
			233	SHVSSMHPD	0.954
			251	MRSNSVRSN	0.996
			254	NSVRSNSVN	0.943
			256	VRSNSVNDN	0.997
			265	LRSRSVNSE	0.851
			268	RSVNSEADI	0.976
			282	SLRASSMSH	0.994
			283	LRASSMSHE	0.982
			285	ASSMSHEMV	0.993
			303	DIGGSSRKF	0.654
			304	IGGSSRKFT	0.993
			312	TRTASQQRN	0.975
			325	DLPPSPESN	0.995
			328	PSPESNGEE	0.991
			429	VEPYSRIDS	0.988
			433	SRIDSAKG-	0.982
EcLsi2_4	545	22	42	FRVISPEDA	0.997
			88	KSRGSKDLL	0.690
			197	WKYLSVEKD	0.848
			220	DEVTSHRFT	0.992
			229	PARMSHVSS	0.990
			232	MSHVSSMHP	0.677
			233	SHVSSMHPD	0.954
			251	MRSNSVRSN	0.996
			254	NSVRSNSVN	0.943
			256	VRSNSVNDN	0.997
			265	LRSRSVNSE	0.851
			268	RSVNSEADI	0.976
			282	SLRASSMSH	0.994

			283	LRASSMSHE	0.982
			285	ASSMSHEMV	0.993
			303	DIGGSSRKF	0.654
			304	IGGSSRKFT	0.993
			312	TRTASQQRN	0.975
			325	DLPPSPESN	0.995
			328	PSPESNGEE	0.991
			429	VEPYSRIDS	0.988
			433	SRIDSAKGV	0.972
EcLsi2_5	447	22	53	FRVISPEDA	0.997
			99	KSRGSKDLL	0.690
			208	WKYLSVEKD	0.848
			231	DEVTSHRFT	0.992
			240	PARMSHVSS	0.990
			243	MSHVSSMHP	0.677
			244	SHVSSMHPD	0.954
			262	MRSNSVRSN	0.996
			265	NSVRSNSVN	0.943
			267	VRSNSVNDN	0.997
			276	LRSRSVNSE	0.851
			279	RSVNSEADI	0.976
			293	SLRASSMSH	0.994
			294	LRASSMSHE	0.982
			296	ASSMSHEMV	0.993
			314	DIGGSSRKF	0.654
			315	IGGSSRKFT	0.993
			323	TRTASQQRN	0.975
			336	DLPPSPESN	0.995
			339	PSPESNGEE	0.991
			440	VEPYSRIDS	0.988
			444	SRIDSAKG-	0.982
EcLsi3_6	518	8	93	GRLLSWRSH	0.965
			236	EDVTSHRFS	0.983
			240	SHRFSPATM	0.993
			245	PATMSHLRA	0.635
			253	ARASSAHPL	0.986
			307	NNSNSNKVT	0.670
			460	AAAISPAAE	0.560
			499	QARRSQHYG	0.985
EcLsi3_7	518	8	93	GRLLSWRSH	0.965
			236	EDVTSHRFS	0.983
			240	SHRFSPATM	0.993
			245	PATMSHLRA	0.635
			253	ARASSAHPL	0.986
			307	NNSNSNKVT	0.670
			460	AAAISPAAE	0.560
			499	QARRSQHYG	0.985
EcLsi3_8	519	9	93	GRLLSWRSH	0.965
			233	EDVTSHRFS	0.983
			237	SHRFSPATM	0.993
			242	PATMSHLRA	0.635

			250	ARASSPLAC	0.980
			255	PLACSDDDP	0.955
			300	NKLDSFHHW	0.837
			446	AAAISPAAE	0.560
			485	QARRSQHYG	0.985
EcLsi3_9	480	22	53	FRVISPEDA	0.997
			99	KSRGSKDLL	0.690
			208	WKYLSVEKD	0.848
			231	DEVTSHRFT	0.992
			240	PARMSHVSS	0.990
			243	MSHVSSMHP	0.677
			244	SHVSSMHPD	0.954
			262	MRSNSVRSN	0.996
			265	NSVRSNSVN	0.943
			267	VRSNSVNDN	0.997
			276	LRSRSVNSE	0.851
			279	RSVNSEADI	0.976
			293	SLRASSMSH	0.994
			294	LRASSMSHE	0.982
			296	ASSMSHEMV	0.993
			314	DIGGSSRKF	0.654
			315	IGGSSRKFT	0.993
			323	TRTASQQRN	0.975
			336	DLPPSPESN	0.995
			339	PSPESNGEE	0.991
			442	VEPYSRIDS	0.988
			446	SRIDSAKGV	0.972
EcLsi1_10	162	2	21	CGAASIYGE	0.971
			31	NKRISQLGQ	0.992

Table S5. Details of predicted sub-cellular location of finger millet silicon transporter proteins predicted by using Wolfpsort, Cello and TargetP servers.

Gene_ID	Wolfpsort[1]	Cello[2]	TargetP[3]
EcLsi1	plasma membrane	plasma membrane	Any other
EcLsi2	plasma membrane	plasma membrane	Secretory
EcLsi6	Endoplasmic reticulum	plasma membrane	Any other
EcLsi2_1	plasma membrane	plasma membrane	Secretory
EcLsi2_2	plasma membrane	plasma membrane	Secretory
EcLsi2_3	plasma membrane	plasma membrane	Secretory
EcLsi2_4	plasma membrane	plasma membrane	Secretory
EcLsi2_5	plasma membrane	plasma membrane	Secretory
EcLsi3_6	plasma membrane	plasma membrane	Secretory
EcLsi3_7	plasma membrane	plasma membrane	Secretory
EcLsi3_8	plasma membrane	plasma membrane	Secretory
EcLsi3_9	plasma membrane	plasma membrane	Secretory
EcLsi1_10	Vacuole	plasma membrane	Secretory

[1]http://wolfpsort.org/, [2]http://cello.life.nctu.edu.tw, [3]www.cbs.dtu.dk/services/TargetP,

Table S6. Primer sequences and their characteristics designed for qRT-PCR analysis of silicon transporter genes predicted by Primer 3 tool.

**Primer**	**sequence primers (5’-3’)**	**Length**	**Start**	**Stop**	**Tm**	**GC%**	**Self**	**Self3**	**product**
qLsi2-F	GCTCTTCACCAACGACACCT	20	345	364	60.25	55.00	2.00	0.00	169bps
qLsi2-R	GAAGGCGATGACGAGGTTC	20	513	494	59.83	55.00	3.00	1.00	
qLsi1-F	CACTGGCACTCTCTGCTCAT	20	487	506	59.75	55.00	3.00	2.00	172bps
qLsi1-R	GGTTCATCGATCCTCCTGAA	20	658	639	56.72	50.00	6.00	3.00	
qLsi6-F	CGTCCCCACCATGTATTACC	20	90	109	57.75	55.00	5.00	3.00	204bps
qLsi6-R	ATCATGACGGTGACGATGAG	20	293	274	57.50	50.00	7.00	1.00	

Table S7. Primer sequences and their characteristics designed for qRT-PCR analysis of genes involved in defense hormone regulation predicted by Primer 3 tool.

**Primer Code**	**qRT-PCR Primers (5'-3')**	**Primer Length**	**Tm**	**GC%**	**Self**	**Self3**	**Start**	**Stop**	**Primers designed from**	**Product Length**
Primers for Salicylic acid										
PAL-F	CCATCGAGCGCGAGGTCAAC	20	63.80	65.00	6.00	2.00	-	-	Conserved sequences	266 bps
PAL-R	GGCGATCTCGGTGCCCTTG	19	63.37	68.42	4.00	0.00	-	-		
Primers for Jasmonic acid										
LOX-F	GTCAAGAACCACTGGAGCGA	20	59.97	55.00	5.00	0.00	355	374	KF813007.2	147 bps
LOX-R	GTCATTGGCGAAGACGATGC	20	59.97	55.00	6.00	2.00	501	482		
Primers for Ethylene										
SAM-F	CGCAAGATCATCATCGACACC	21	59.48	52.38	6.00	0.00	-	-	Conserved sequences	394 bps
SAM-R	CCACCTCCCAGGTGAAGTC	19	59.32	63.16	7.00	3.00	-	-		
Internal Control Actin										
EcActin-F	AAGTACCCAATCGAGCATGG	20	60.0	50.00	4.00	2.00	83	103	HE800188.1	247 bps
EcActin-R	CAGTTGTACGCCCACTAGCA	20	59.9	55.00	4.00	0.00	329	349		
